# Phase Transition Thermodynamics of Organic Semiconductors 1,3-Bis(9H-carbazol-9-yl)benzene, 1,3,5-Tri(9H-carbazol-9-yl)benzene, 1,3,5-Tris(diphenylamino)benzene, and 1,3,5-Tris[(3-methylphenyl)phenylamino]benzene

**DOI:** 10.3390/molecules31091435

**Published:** 2026-04-26

**Authors:** Airat A. Notfullin, Dmitrii N. Bolmatenkov, Andrey A. Sokolov, Ilya S. Balakhontsev, Mansur B. Khisamiev, Boris N. Solomonov, Mikhail I. Yagofarov

**Affiliations:** Department of Physical Chemistry, Kazan Federal University, Kremlevskaya Str. 18, 420008 Kazan, Russia; dmnbolmatenkov@kpfu.ru (D.N.B.); andasokolov@kpfu.ru (A.A.S.); isbalakhoncev@kpfu.ru (I.S.B.); mabkhisamiev@kpfu.ru (M.B.K.); miiyagofarov@kpfu.ru (M.I.Y.)

**Keywords:** organic semiconductors, vapor pressure, melting, vaporization, sublimation, solution calorimetry, fast scanning calorimetry, polymorphism, phase transition

## Abstract

Organic light-emitting diode (OLED)-based devices continue to grow rapidly in popularity. This work presents a comprehensive thermodynamic study of four nitrogen-containing organic semiconductors: 1,3-bis(9H-carbazol-9-yl)benzene (mCP), 1,3,5-tri(9H-carbazol-9-yl)benzene (TCB), 1,3,5-tris(diphenylamino)benzene (TDAB), and 1,3,5-tris[(3-methylphenyl)phenylamino]benzene (m-MTDAB). A self-consistent set of phase-change thermodynamic parameters in a wide temperature range was obtained using several independent experimental and computational techniques. Vapor pressure measurements above the liquid and crystalline phases of the compounds under study were carried out using the thermogravimetry–fast scanning calorimetry method. Based on the temperature dependence of the measured vapor pressures, vaporization and sublimation enthalpies were derived. Differential scanning calorimetry was employed to determine the heat capacities of the condensed phases and the melting parameters of the studied compounds, as well as to investigate the polymorphism of TCB. Solution calorimetry was used to determine the fusion enthalpies of the compounds at 298.15 K. The obtained values were additionally compared with the literature data and calculated estimates. The results of this study may be used to predict properties for compounds with similar molecular structures.

## 1. Introduction

Nowadays, electronic devices based on organic light-emitting diodes (OLEDs) have a leading position on the market [1]. They combine flexibility, lightness, low cost, and high performance [2]. In addition, a fine variation in their chemical structure allows one to tune their optical properties [3].

Several types of organic molecules are used in OLEDs: aromatic molecules, conjugated polymers, complexes, or their combination [4,5,6,7,8]. The type defines performance (such as quantum yield, transparency, brightness), preparation conditions, and production cost. For relatively low molecular weight organic compounds, physical vapor deposition (also known as physical vacuum deposition and dry deposition) currently remains a preferable production method for electronic devices [6]. The benefits of physical vapor deposition include the capability to produce multilayer and doped OLEDs; the creation of highly uniform, ultrastable amorphous phases; accurate layer thickness control for precise property adjustment; and the low cost and high purity of the resulting coatings [6].

Production, utilization, and understanding of key properties of OLEDs are strongly associated with the knowledge of the phase transition thermodynamic parameters of the constituent compounds. In particular, the data on the temperature dependence of the vapor pressure of OLED components substantially facilitates the control of the temperature regime during evaporation. The vapor pressure defines the deposition rate [8], which, in its turn, influences the film microstructure and electroluminescent properties. When several components are deposited at once, sublimation/vaporization thermodynamics define their ratio in the resulting film. The relative stabilities of the crystal, liquid, and glassy states over a wide temperature range (assessed based on the thermodynamic parameters of melting/crystallization [9] and fictive temperature derived from DSC [10,11]) define the long-term stability (crystallization and relaxation tendency) and performance of metastable amorphous film-based organic electronics [9,10,12]. Tuning the substrate temperature during the physical vapor deposition process can influence the crystallinity degree and the morphological characteristics of the thin film and, thus, tailor the functional properties and minimize the production cost [13]. Nowadays, the deposition regime is usually optimized empirically [13]; however, the optimization based on the saturated vapor pressures and their temperature dependences is more effective [14].

Thus, the data on the vapor pressures above crystalline and liquid phases, sublimation, vaporization, and fusion enthalpies over a wide temperature range are of interest for organic semiconductors. However, their low accessibility is caused by low volatility, high melting temperatures, and purification issues. Recently, most results in this field were obtained by the group of José Costa from the University of Porto [15,16], group of Juan Mentado-Morales from the Universidad del Mar [17], and our laboratory [18,19,20]. However, some discrepancies are found between the data obtained by different groups, particularly at 298.15 K [18,19,20]. This underscores the need for additional data verification. The feature of our studies is the application of the solution calorimetry (SC) method to access enthalpy of fusion directly at 298.15 K, which makes indirect verification of the values obtained possible using Kirchoff’s Law of Thermochemistry [21]. In addition, we apply fast scanning calorimetry to attain extremely low vapor pressures [22,23,24] and the heat capacity of the supercooled liquid state for fast-crystallizing compounds [25].

**Figure 1:**
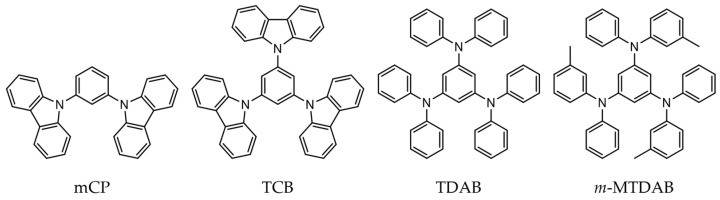
Chemical structures of compounds studied in this work.

In this work, we continued our studies of phase transition thermodynamics for organic semiconductors widely used in OLEDs production and focused on four compounds: 1,3-bis(9H-carbazol-9-yl)benzene (mCP), 1,3,5-tri(9H-carbazol-9-yl)benzene (TCB), 1,3,5-tris(diphenylamino)benzene (TDAB), and 1,3,5-tris[(3-methylphenyl)phenylamino]benzene (*m*-MTDAB) (Figure 1). Their thermodynamic properties were partially characterized previously [15,16,17,26]; however, some gaps in their condensed phase heat capacities, vaporization and sublimation enthalpies still exist. Furthermore, for TCB, the melting temperatures and fusion enthalpies reported in different papers [16,26,27,28,29] are scattered by 130 K and 30 kJ·mol^−1^ (~50%), respectively. Filling the above gaps and resolving the controversies requires the combined experimental studies, particularly employing non-conventional approaches to clarify the possible effects of crystal polymorphism and decomposition at elevated temperatures.

In this respect, we used differential scanning calorimetry (DSC), fast scanning calorimetry (FSC), and solution calorimetry, together with quantum chemical and statistical thermodynamics-based computations to derive the phase transition thermodynamic characteristics over a wide temperature range between 298.15 K and melting temperatures of compounds (*T*_m_).

Structural motifs in the compounds under study are common for other nitrogen-containing organic semiconductors studied previously by our group [18,20] and elsewhere [15,16,17,26,30,31,32]. The set of phase transition thermodynamic data on these compounds was analyzed by applying simple additivity principles, which confirmed the mutual consistency of the respective properties and allowed the estimation of these values for larger and less volatile molecules of similar structures [3].

## 2. Results

In this section, we separately discuss the melting properties of compounds under study (Section 2.1), particularly the polymorphism of TCB (Section 2.1.1), computed ideal gas thermodynamic properties (Section 2.2), condensed phase heat capacities (Section 2.3), and results of vapor pressure measurements (Section 2.4).

### 2.1. Fusion Thermochemistry at T_m_

The experimental and literature values of melting (onset) temperatures and fusion enthalpies $\Delta_{cr}^{l}H(T_{m})$ of mCP, TCB, TDAB, and *m*-MTDAB are displayed in Table 1.

For mCP, one can see some inconsistency in both melting points and fusion enthalpies. The two reported *T*_m_ values [34,35] deviate notably from others. In the case of ref. [34], this discrepancy can be explained by the fact that the melting temperature was taken as the peak temperature instead of the standard onset temperature. For ref. [35], it was not possible to verify how this melting point was determined, as the corresponding DSC heating curves were not provided in the manuscript. The other literature values show sufficient consistency with *T*_m_ measured in this study. Regarding the fusion enthalpy, the value reported in ref. [17] is ~4 kJ mol^−1^ higher than those determined in this work and in ref. [16]. The reason for this difference is unclear, considering the excellent agreement between the heat capacities of the crystalline phase of mCP at 298.15 K measured in the present work and in ref. [17] (see below).

A certain spread in melting temperatures and enthalpies is also observed for TDAB, whereas the literature and experimental values of *T*_m_ and $\Delta_{cr}^{l}H(T_{m})$ for *m*-MTDAB are in good agreement.

The data on TCB are more complicated. Studies [26,28] mention the existence of two polymorphic modifications of the compound with melting points around 548 K (crystal B) and 600 K (crystal A). Study [16] reports a melting point for TCB of 597.5 K. Meanwhile, Refs. [27,29] provide significantly different *T*_m_ values of 467.15 K and 570.15 K, respectively. These discrepancies motivated a detailed reinvestigation of this compound’s polymorphism. Our study successfully identified three distinct polymorphic forms of TCB, with melting points at 544.9 K (crystal B), 565.6 K (crystal C), and 599.2 K (crystal A). All three polymorphs were characterized using X-ray powder diffraction (Figure S1).

A comparison of the literature *T*_m_ values with our results shows better agreement for polymorph A than for crystals B and C. The fusion enthalpies measured in this work align well with the results presented in ref. [26] and, to a lesser extent, with the values reported in ref. [28]. The fusion enthalpy of polymorph A reported in ref. [16] is notably underestimated compared to the remaining data. Other findings on the polymorphism of TCB are given in the next section.

#### Polymorphism of TCB

Crystallization of TCB from the melt proved to be a stochastic process. Identical experimental conditions often yielded different polymorphic forms. For this reason, the following section will primarily address the signs of each polymorph crystallization, with secondary attention given to the crystallization conditions themselves.

In our experiments during the polymorphism study, the heating rates on the differential scanning calorimeter varied from 10 to 50 K min^−1^. In most cases, a cooling rate of 50 K min^−1^ was used after melting, under which no crystallization was observed for any of the polymorphs. With a moderately slow cooling rate (10 K min^−1^), we recorded a crystallization peak for polymorph A at 494 K, indicating that crystalline phase A can nucleate at high temperatures. In all other cases, crystallization peaks appeared upon heating the amorphous glass phase obtained by rapid cooling (50 K min^−1^) of the melt.

**Figure 2:**
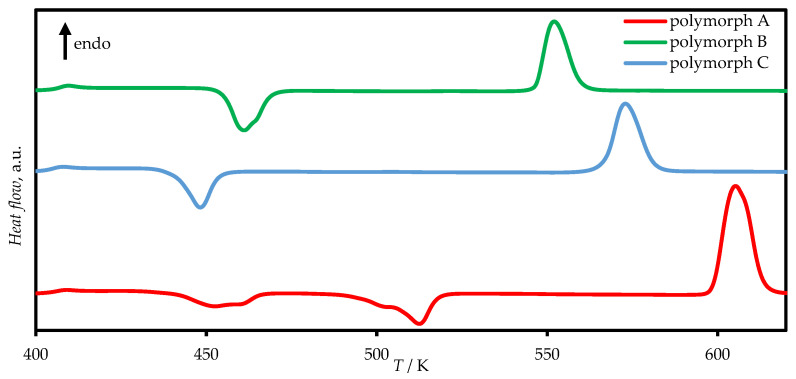
Typical DSC scans of the crystallization and melting of TCB polymorphs at the heating rate of 50 K min^−1^.

During heating of the glass phase, the formation of polymorph A exhibits several exothermic effects on the same heating scan (Figure 2). It is most likely that the peaks correspond to the crystallization of a less stable polymorphic modification with subsequent polymorphic transitions resulting in the formation of crystal A.

Crystal B formed under relatively slow heating (10 K min^−1^) or after the glass was held at room temperature for 1–2 days (Figure 2). It could be suggested that the nucleation process for crystalline phase B occurs at lower temperatures, including those below the glass transition (*T*_g_ ≈ 393 K at a scanning rate of 10 K min^−1^).

No clear relationship was identified between the crystallization conditions and the probability of polymorph C forming. A more detailed analysis of the crystallization processes of the TCB polymorphs is planned for future studies.

Using the melting data from DSC scans, a $\Delta_{cr}^{l}G(T)$–*T* diagram (Gibbs free energy of fusion versus temperature) was plotted for all three polymorphs. A more common, though in this case less illustrative, version of a semi-schematic *G*–*T* diagram for the liquid phase and the polymorphs of TCB is provided in the Supplementary Materials (Figure S7). To account for the temperature dependence of the Gibbs free energies of fusion for polymorphs B and C, the heat capacity values measured for crystalline form A were used as approximate substitutes. Further refinement of the data is planned once the reproducible preparation of the metastable polymorphs is optimized.

**Figure 3:**
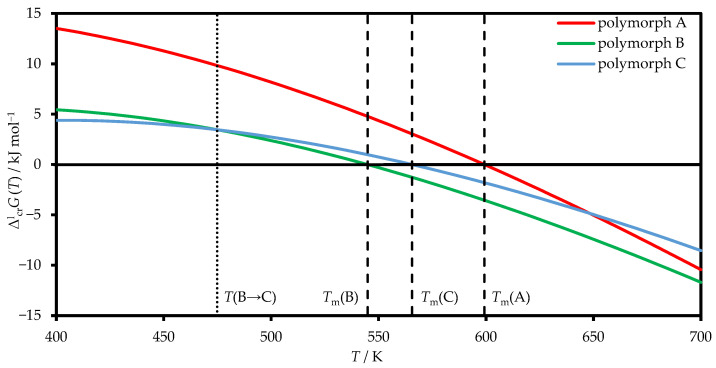
Gibbs free energy of fusion versus temperature diagram of TCB polymorphs. The temperatures of the corresponding transitions are indicated by dashed lines.

As can be seen in Figure 3, polymorph A shows the highest stability across the temperature range up to its melting point. Polymorphs B and C display an enantiotropic polymorphic transition from B to C at approximately 475 K. Thus, when determining the thermodynamic properties of polymorph A below its melting point, no change in the phase composition is observed during the study.

The X-ray powder diffraction results for polymorph A from ref. [28] align with those obtained in this work (Figure S1). However, based on our measurements, the diffractogram previously reported in that study for crystalline phase B actually corresponds to polymorph C. The formation of polymorph C is possible under the same conditions in which polymorph B forms in ref. [28], which, as noted earlier, is due to the stochastic nature of TCB crystallization.

### 2.2. Ideal Gas Heat Capacities

**Figure 4:**
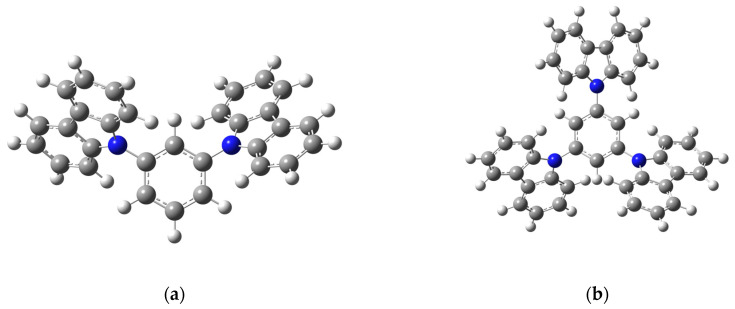

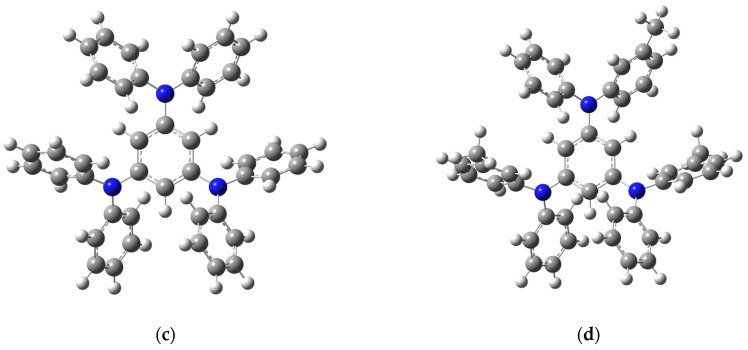
Optimized geometries of mCP (**a**), TCB (**b**), TDAB (**c**), and *m*-MTDAB (**d**) in the gas phase obtained at the B3LYP/6-31+G(d,p) theory level. Blue atoms correspond to nitrogen, gray—carbon, white—hydrogen.

The optimized geometries of mCP, TCB, TDAB, and *m*-MTDAB are shown in Figure 4.

No calculations for mCP and TCB in the gas state were found in the literature, but the structural and thermodynamic properties of TDAB and *m*-MTDAB were previously reported by Costa et al. [15]. They used the same level of theory as in this work (B3LYP/6-31+G(d,p)), and therefore a comparison of the results obtained would be beneficial.

The structural properties and heat capacities at 298.15 K of mCP, TCB, TDAB, and *m*-MTDAB in the ideal gas state determined in this work and presented by Costa et al. are collected in Table 2.

The electronic energies of TDAB and *m*-MTDAB in this work are slightly lower than in ref. [15]. In most cases, the bond lengths and valence angles in these molecules agree within 0.02 Å and 0.3 degrees, respectively. The exceptions are some valence angles in *m*-MTDAB that differ by 1–1.5 degrees, indicating that phenylenediamine and *m*-tolylphenylamino groups are located slightly closer to each other in this work than in the structure obtained in ref. [15]. Higher discrepancies, reaching 10–15 degrees, are observed for the dihedral angles in both molecules. This means that the phenylenediamine groups in the geometries of TDAB and *m*-MTDAB determined in this work are more planar and are more parallel to the plane of the central benzene ring than in ref. [15]. Despite the above differences, the structures of TDAB and *m*-MTDAB obtained in this work and by Costa et al. are practically identical and have the same conformation of substituents relative to each other.

The main disagreement between the results of this work and those presented by Costa et al. lies in the heat capacities of the ideal gas phase. The values of *C*_p,m_(g, 298.15 K) for TDAB and *m*-MTDAB are 18 J K^−1^ mol^−1^ (2–3%) lower in this work than reported in ref. [15]. There are two main reasons for this discrepancy: the differences in the scaling factors and the lack of internal rotation treatment in ref. [15]. Using the full set of frequencies for TDAB calculated in this work (even those corresponding to internal rotations) together with the scaling factors implemented by Costa et al. gives a value of *C*_p,m_(g, 298.15 K) = 641.7 J K^−1^ mol^−1^, which is very close to 642.8 J K^−1^ mol^−1^ from ref. [15]. This confirms that the observed discrepancy arises not from differences in the obtained structures but from the approaches used to process the calculation results.

Similar deviations were observed earlier for several compounds (TPB, TPD, DDP, and *p*-TTP; Figure S7) investigated by our research group [18,20] and Costa et al. [15]. All these molecules contain two diphenylamine groups, either methylated or non-methylated, and the differences in the ideal gas heat capacities at 298.15 K were in the range of 12–13 J K^−1^ mol^−1^. Considering that for three such groups in TDAB and *m*-MTDAB the deviation is 18 J K^−1^ mol^−1^, one can conclude that the lack of internal rotation treatment and the use of a single scaling factor for vibrational frequencies introduces an error of 6 J K^−1^ mol^−1^ per phenylenediamine group.

### 2.3. Condensed Phase Heat Capacities

**Figure 5:**
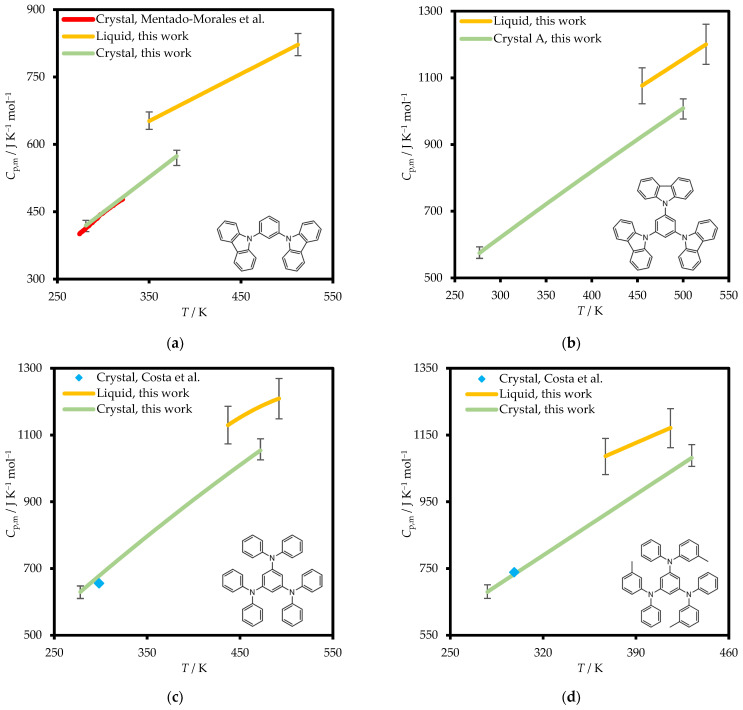
Condensed phases heat capacities of mCP (**a**), TCB (**b**), TDAB (**c**), and *m*-MTDAB (**d**) measured in this work and in refs. [15] (Costa et al.) and [17] (Mentado-Morales et al.). Error bars represent standard deviation of the experimental heat capacities.

The heat capacities of condensed phases of mCP, TCB, TDAB and *m*-MTDAB measured in this work are shown in Figure 5.

The experimental heat capacity values of the condensed phases measured in this work were described as a polynomial function of temperature:

(1) $$C_{p,m}(T)/(J\;K^{-1}\;mol^{-1})=a+b\cdot (T/K)+c\cdot {\left(T/K\right)}^{2}$$

The parameters of Equation (1) are presented in Table 3. Heat capacities at temperatures outside the measurement ranges necessary for the adjustment of the thermodynamic functions (Section 3) were obtained by linear extrapolation. The uncertainty of the extrapolated heat capacity values was mainly contributed by the experimental error (see Equation (S1)) due to the wide temperature range of measurement and high sampling frequency of DSC (i.e., amount of *C*_p,m_ datapoints). Deviations from the linearity would introduce more significant extrapolation error, which might especially contribute to the heat capacity integrals involving *C*_p,m_(l). However, the validity of the linear extrapolation of *C*_p,m_(l) temperature dependence down to 298.15 K for organic non-electrolytes was repeatedly demonstrated in our previous works [18,19,20,21] and is confirmed in this work by the comparison between the fusion enthalpies at 298.15 K obtained by solution calorimetry and Kirchhoff’s Law of Thermochemistry.

We compared experimentally determined heat capacities for mCP, TDAB, and *m*-MTDAB crystals at 298.15 K with the previously reported literature values [15,17] (Table 4). Our results for mCP and *m*-MTDAB showed excellent consistency with the literature data, deviating by less than 1%. For TDAB, the discrepancy was slightly larger at approximately 3%; however, it is still within the combined uncertainty of both this work and ref. [15].

### 2.4. Saturated Vapor Pressures

**Figure 6:**
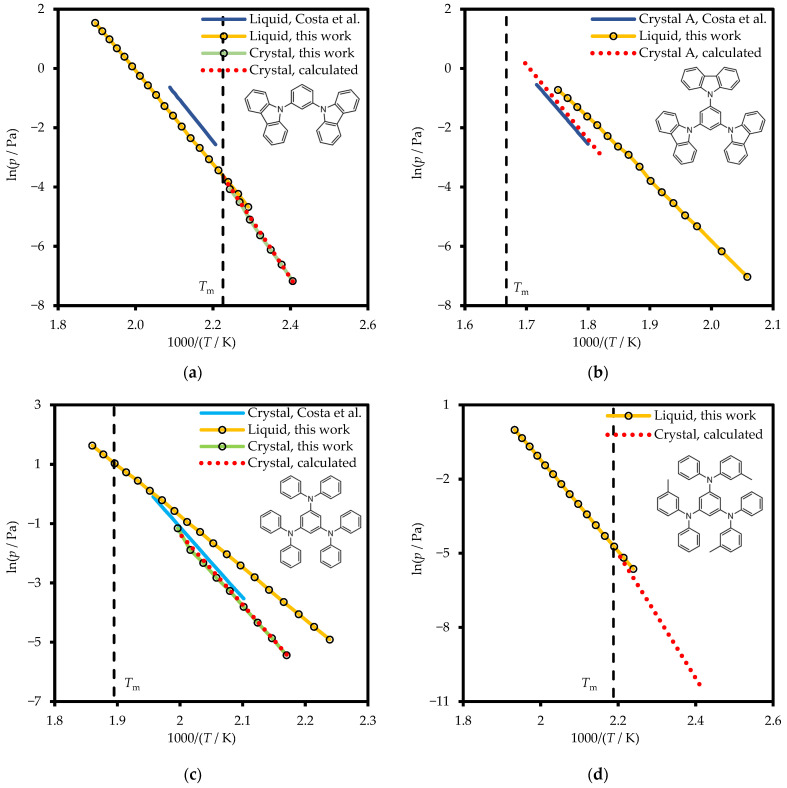
Vapor pressures of mCP (**a**), TCB (**b**), TDAB (**c**), and *m*-MTDAB (**d**) measured in this work and in refs. [15] (light blue) and [16] (dark blue).

The literature and experimental vapor pressures over the crystalline phases of mCP and TDAB and liquid (including supercooled liquid) phases of mCP, TCB, TDAB, and *m*-MTDAB are presented in Figure 6 and in Table S8 of the Supplementary Materials.

Temperature dependences of saturated vapor pressures *p* were fitted using the Clarke–Glew equation:

(2) $$ln(p/Pa)=ln(p(T_{0})/Pa)-\frac{\Delta_{cr/l}^{g}H(T_{0})}{R}(\frac{1}{T}-\frac{1}{T_{0}})+\frac{\Delta_{cr/l}^{g}C_{p,m}}{R}(\frac{T_{0}}{T}-1+ln(\frac{T}{T_{0}}))$$

where *T*_0_ is the reference temperature equal to 298.15 K or *T*_m_ (melting point), $\Delta_{cr/l}^{g}H(T_{0})$—sublimation/vaporization enthalpy at *T*_0_, and $\Delta_{cr/l}^{g}C_{p,m}$—isobaric molar heat capacity difference in gaseous and crystalline/liquid phases.

The Clarke–Glew equation does not take into account the temperature dependence of the heat capacities and therefore their differences. So, the average value of $\Delta_{cr/l}^{g}C_{p,m}$ for the temperature range was calculated as follows:

(3) $$\Delta_{cr/l}^{g}C_{p,m}=\frac{\int_{T_{1}}^{T_{2}}\Delta_{cr/l}^{g}C_{p,m}(T)dT}{T_{2}-T_{1}}$$

The parameters of the Clarke–Glew equation (Equation (2)) are compiled in Table 5 and Table S10. For all compounds, vapor pressures over the crystalline phase were additionally calculated based on evaporation and fusion data. The calculation details are provided in Section S5 (Table S9) of the Supplementary Materials. As can be seen in the figure, both mCP and TDAB calculated values show good consistency with the experimental results.

The previous literature reported vapor pressure data for liquid mCP and the crystalline phases of TCB (polymorph A) and TDAB (Figure 6) measured using a quartz crystal microbalance Knudsen effusion method [15,16]. As shown in the figure, a significant discrepancy is observed between the literature values and experimental vapor pressures for liquid mCP, differing by a factor of approximately 2.2. Nevertheless, the slopes of the ln*P* vs. 1000/*T* curves are in good agreement.

A comparison of vapor pressures over crystalline TDAB shows a smaller discrepancy. The difference between the literature data and our measurements is ~30%, which lies within the combined experimental uncertainty of the techniques employed in ref. [15] and this study.

TCB presents a distinct situation compared to mCP and TDAB. First, it should be noted that the comparison was made using vapor pressures calculated from TCB evaporation and melting data. While the vapor pressure values themselves are moderately close (differing by 13–30% depending on temperature), the slopes of the curves differ significantly. Notably, the difference between the sublimation enthalpies derived from these vapor pressure–temperature dependences (213.7 − 119.9 = 13.8 kJ mol^−1^) is almost exactly the same as the difference between the melting enthalpies (56.3 − 41.7 = 14.6 kJ mol^−1^) measured in this work and in ref. [16]. Incomplete crystallinity of the sample from ref. [16] could be proposed as an explanation for this discrepancy.

As in the previous cases, no systematic difference was observed between the results obtained by the two different experimental methods: FSC in this and previous works [18,20] and the Knudsen effusion method from the literature [15,16].

## 3. Discussion

This section focuses on verifying the experimental data obtained, for the reasons mentioned earlier in the introduction. The fusion enthalpies at 298.15 K derived from two independent methods are compared (Section 3.1). The additivity of heat capacities is examined for the ideal gas and condensed phases of the studied compounds (Section 3.2 and Section 3.3, respectively). The vaporization and sublimation enthalpies are compared with the literature values (Section 3.4) along with DSC and SC data for mCP and TDAB.

To compare the data obtained in this study with those reported in the literature, all values must be recalculated to the same temperature. Kirchhoff’s Law of Thermochemistry was used to correct the phase transition enthalpies to the temperature of interest for further comparison:

(4) $$\Delta_{\mathrm{phase}\;1}^{\mathrm{phase}\;2}H(T_{2})=\Delta_{\mathrm{phase}\;1}^{\mathrm{phase}\;2}H(T_{1})+\int_{T_{1}}^{T_{2}}\Delta_{\mathrm{phase}\;1}^{\mathrm{phase}\;2}C_{p,m}(T)dT$$

where $\Delta_{\mathrm{phase}\;1}^{\mathrm{phase}2}H$ is a phase transition enthalpy and $\Delta_{\mathrm{phase}\;1}^{\mathrm{phase}\;2}C_{p,m}$ is a heat capacity difference in two phases obtained in this work from DSC and FSC measurements and/or computations.

### 3.1. Fusion Thermochemistry at 298.15 K

For additional validation of our results, we carried out solution calorimetry experiments. Using solution enthalpy data and Hess’s law, the fusion enthalpies at 298.15 K were calculated:

(5) $$\Delta_{cr}^{l}H^{A}(298.15\;K)=\Delta_{soln}H^{A/S}(\mathrm{cr},\;298.15\;K)-\Delta_{soln}H^{A/S}(l,\;298.15\;K)$$

where $\Delta_{cr}^{l}H^{A}$—fusion enthalpy of compound A, $\Delta_{soln}H^{A/S}$—solution enthalpy of compound A in solvent S, cr—crystal, and l—liquid.

Previously, we demonstrated that the solution enthalpies of compounds in the liquid phase can be estimated using the “like dissolves like” principle for many solvent–solute systems [38], including those with benzene as a solvent and organic semiconductors as solutes [18,19,20]. This enables calculation of fusion enthalpies for the studied compounds at 298.15 K from the solution enthalpies of their crystalline phases.

Fusion enthalpies at 298.15 K, calculated using Hess’s law (based on solution calorimetry data, Equation (5)) and Kirchhoff’s Law (based on DSC data, Equation (4)), were then compared with each other and with the literature (Table 6).

The values derived in the present work are consistent for each compound under study when considering the combined uncertainties of both methods. Fusion enthalpies at 298.15 K for these compounds have also been reported previously in the literature [15,16]. The authors used Kirchhoff’s Law of Thermochemistry to adjust their measurements from *T*_m_ to 298.15 K. In each case, these values are significantly overestimated compared to the results obtained in the present work. This discrepancy is due to the use of a constant $\Delta_{cr}^{l}C_{p,m}$ value (50 ± 10 J K^−1^ mol^−1^) that is significantly (2–3 times) lower than the actual heat capacity difference. This finding emphasizes the importance of performing experimental measurements of heat capacities over a wide temperature range or developing new heat capacity calculation approaches that account for temperature dependence as well as highlights the advantages of the solution calorimetry approach for direct estimation of $\Delta_{cr}^{l}H(T_{0})$.

### 3.2. Verification of Ideal Gas Heat Capacities

Analysis of new data obtained on the compounds under study makes it possible to derive additive contributions for structural groups in the molecules of organic semiconductors. For example, we can consider the ideal heat capacity of benzene at 298.15 K as 82.4 J K^−1^ mol^−1^ [39] and use the values of *C*_p,m_(g, 298.15 K) for DDP, TDAB, mCP, and TCB calculated in this (Figure 1) and previous [18,20] (Figure S7) works (Table S19). This allows us to establish the contributions of the carbazolyl and phenylenediamine groups to the ideal heat capacity at 298.15 K. The average value for the carbazolyl group contribution was calculated to be 166.7 ± 0.3 J K^−1^ mol^−1^, and that of the diphenylamine group was 180.6 ± 0.4 J K^−1^ mol^−1^. As can be seen, the deviations from the average values do not exceed the uncertainties of the heat capacity calculations. This is surprising, considering that the groups in question have different steric hindrances when rotating in different compounds and are sometimes even located at different positions on the benzene ring. Apparently, these differences do not significantly affect the contribution of these groups to *C*_p,m_(g), which means that the obtained data on the compounds studied can be used to predict the heat capacities of other similar organic semiconductors in the ideal gas phase [3].

### 3.3. Additivity of Condensed Phase Heat Capacities

The number of structurally similar organic semiconductors studied to date enables the mutual verification of their heat capacity data. To check the consistency of the obtained data, we combined the experimental *C*_p,m_(cr, 298.15 K) and *C*_p,m_(liq, 298.15 K) (Table 7) values for mCP, TCB, TDAB, and *m*-MTDAB (studied in this work) with those for DDP, *p*-TTP, TPB, TPD, and CBP (reported previously [18,20]).

First, we calculated the group contributions of the benzene ring and the methyl, carbazolyl, and diphenylamino groups based on experimental data for mCP, CBP, DDP, TPB, *p*-TTP, and TPD (Table S20). Then, using these contributions, we calculated the heat capacities of the liquid and crystalline phases of TCB, TDAB, and *m*-MTDAB at 298.15 K (Table 7). The differences between the calculated and experimental values for the condensed phases were within 5%. This confirms the reliability of the experimental data obtained and demonstrates the predictive utility of these group contributions for related compounds.

Additionally, we calculated the heat capacities of both the liquid and crystalline phases of these compounds using group additivity-based prediction methods [40,41,42] and compared these values with our results. As can be seen in Table 7, both calculation methods show good agreement with our experimental values of liquid heat capacities, with deviations of less than 10% (4% on average for both methods). For the crystalline phases of organic semiconductors, the heat capacities estimated by ref. [40] also show good accuracy, with an even lower maximum error of 7%. The values calculated using the method presented in ref. [41] and updated in ref. [42], on the other hand, showed good agreement only for mCP, CBP, and TCB. The calculated heat capacities of DDP, *p*-TTP, TPB, TPD, TDAB, and *m*-MTDAB are 14–19% higher than the experimental ones. It seems that the contribution to the “tertiary sp^3^ nitrogen” for crystalline heat capacity presented in ref. [41] is overestimated. It should be noted that the value of this group is considered to be tentative in ref. [41] because of the small number of literature data points available to establish it.

### 3.4. Vaporization and Sublimation Enthalpies

In refs. [15,16], the vaporization enthalpies for mCP and sublimation enthalpies for TDAB and TCB were derived from vapor pressure data at the mean temperatures of the effusion ranges and at 298.15 K. In ref. [17], $\Delta_{l}^{g}H$ for mCP was determined using the thermogravimetry method based on the dependence of the sample’s mass loss rate during evaporation on temperature. Subsequently, this $\Delta_{l}^{g}H$ value was used to calculate the sublimation enthalpy at the melting point and at 298.15 K. These literature values are compiled in Table 8 for comparison with the results of the present study.

To enable data comparison, the measured vaporization and sublimation enthalpies were recalculated (fifth column of Table 8) to the temperatures at which the literature values were reported (third column of Table 8). The literature and experimental sublimation enthalpies compiled in the table show good agreement, with the exception of the previously mentioned TCB sublimation enthalpies (see Section 2.4 on saturated vapor pressures) and $\Delta_{cr}^{g}H(298.15\;\;K)$ of TDAB. At the same time, the sublimation enthalpies for TDAB at elevated temperatures are in excellent agreement.

The discrepancy in the 298.15 K values most likely stems from the fact that the temperature dependencies of *C*_p,m_(g) and *C*_p,m_(cr) were not accounted for in ref. [15]. While the difference in heat capacities between the gaseous and crystalline phases is often negligible, its contribution can become significant when recalculating sublimation enthalpies from high temperatures, as in the case of TDAB. This observation further highlights the necessity of experimental measurements and calculations of heat capacities for organic compounds across a wide temperature range.

Furthermore, the vaporization enthalpies for mCP reported in ref. [17] at 600 K and ref. [16] at 466 K agree well with the values recalculated to these temperatures from the present work. A much poorer agreement is observed for the literature vaporization enthalpies that were either recalculated to different temperatures using an empirical $\Delta_{l}^{g}C_{p,m}$ value or calculated via Hess’s Law using sublimation and fusion data. In the case of enthalpies derived via Hess’s Law, the deviations are also likely caused by the use of empirical values for $\Delta_{cr}^{g}C_{p,m}$ and/or $\Delta_{cr}^{l}C_{p,m}$ used for the temperature corrections of sublimation and fusion enthalpies.

It is also worth comparing the melting enthalpies of mCP and TDAB at 298.15 K with the differences between their sublimation and vaporization enthalpies at this temperature. These differences are 6.1 kJ mol^−1^ and 17.8 kJ mol^−1^ respectively, showing moderate agreement with the data obtained by DSC and SC. This finding provides further validation of the data obtained in the present work.

## 4. Materials and Methods

In this section, we provide general information about the experimental techniques used in this work. More information regarding the details of the experiments are provided in the Supplementary Materials (Sections S3, S4, S6 and S7).

### 4.1. Materials

Before the experiments, commercial samples of mCP (1,3-bis(9H-carbazol-9-yl)benzene; CAS No. 550378-78-4; Hotspot Biotechnology, Weifang, China), TCB (1,3,5-tri(9H-carbazol-9-yl)benzene; CAS No. 148044-07-9; Hotspot Biotechnology, Weifang, China), TDAB (1,3,5-tris(diphenylamino)benzene; CAS No. 126717-23-5; Hotspot Biotechnology, Weifang, China) and *m*-MTDAB (1,3,5-tris[(3-methylphenyl)phenylamino]benzene; CAS No. 138143-23-4; Hotspot Biotechnology, Weifang, China) were purchased and purified by sublimation under reduced pressure. Their final purities were measured by HPLC. The characterization of the samples and auxiliary compounds used as calibration standards are provided in Table S1 of the Supplementary Materials.

### 4.2. Differential Scanning Calorimetry

Heat capacities of crystalline and liquid (including supercooled liquid) phases, melting temperatures and fusion enthalpies of the studied compounds were measured using a power compensation differential scanning calorimeter Perkin Elmer DSC 8500 (Perkin Elmer, Shelton, Connecticut, USA). Heat capacity measurements were performed at a scanning rate of 10 K min^−1^ along with melting temperatures and enthalpies of mCP, TDAB, and *m*-MTDAB. For TCB, scanning rates varied from 10 K min^−1^ to 50 K min^−1^ (see discussion). All experiments were carried out in a dynamic nitrogen atmosphere (flow rate 30 mL min^−1^). To exclude the possibility of decomposition, repeated heating and cooling scans were performed for each sample. No changes were observed in the experimental results. The results are shown in Figure 5 and Tables S2–S4.

### 4.3. Fast Scanning Calorimetry

Flash DSC1 (Mettler Toledo, Greifensee, Switzerland) and Flash DSC2+ (Mettler Toledo, Greifensee, Switzerland) fast scanning calorimeters were used to measure supercooled liquid phase heat capacities and saturated vapor pressures above liquid (including supercooled liquid) and crystalline phases of mCP, TCB, TDAB and *m*-MTDAB. The thermogravimetry–fast scanning calorimetry method was applied for vapor pressure measurements [24]. Experiments were performed under a nitrogen atmosphere using Flash DSC1 and under an argon atmosphere using Flash DSC2+ with a gas flow rate of 40 mL min^−1^ in both cases. Standard conditioning and correction procedures were performed for the UFS1 chip sensors according to the manufacturer’s recommendations. Calibration was conducted using biphenyl, benzoic acid, and anthracene as standards [45].

Figure 5 and Figure 6 and Figures S2 and S3 and Tables S5–S8 present the experimental values of heat capacities and vapor pressures.

### 4.4. Solution Calorimetry

The solution enthalpies of mCP, TCB, TDAB and *m*-MTDAB in benzene were measured at 298.15 K using a TAM III precision solution calorimeter (SC) (TA Instruments, New Castle, Delaware, USA). For each experiment, a 40–90 mg sample was dissolved in 100 mL of benzene. The absence of concentration dependence in the results confirmed infinite dilution conditions. The measured values and experimental procedure are presented in Tables S11–S12 and Section S6 of the Supplementary Materials.

### 4.5. Computations

The heat capacities of mCP, TCB, TDAB, and *m*-MTDAB in the ideal gas state between 200 and 600 K were calculated by means of quantum chemistry and statistical thermodynamics.

First, the lowest-energy conformers of the molecules under study were found by using the Global Optimizer Algorithm (GOAT) implemented in the ORCA 6.0 software package [46] with the semiempirical GFN2-xTB method [47]. The obtained structures were optimized at the B3LYP/6-31+G(d,p) theory level with the addition of Grimme’s dispersion correction and Becke–Johnson damping (D3BJ) [48].

Then, the vibrational frequencies were calculated using the optimized geometries of mCP, TCB, and TDAB. Those of them corresponding to the internal rotations were identified according to Ayala [49] and excluded from further calculations. The remaining frequencies were scaled by 0.9795 below 2000 cm^−1^ and 0.9566 above 2000 cm^−1^ as recommended in ref. [50].

1-D hindered rotor approximation (1-DHR) [51] was implemented to determine the contributions of the internal rotations to the ideal gas heat capacities of mCP, TCB, and TDAB. For further details on the calculation procedure, see Supplementary Materials.

The use of the 1-DHR model and separate scaling factors for low- and high-frequency vibrational modes results in an average absolute percentage deviation σ_r_ of 1.5% at 300 K and 1% at 600 K [52].

For *m*-MTDAB, only the optimized structure was determined using the quantum chemical methods. Its heat capacities in the ideal gas state were calculated based on the values of *C*_p,m_(g, *T*) for TDAB by adding the contributions of three methyl groups. It was previously shown [20] that the difference between the ideal gas heat capacities of toluene and benzene is almost the same (21.3 J K^−1^ mol^−1^) as that for methylated and non-methylated organic semiconductors of a more complex structure (for DDP and *p*-TTP—21.4 J K^−1^ mol^−1^, for TPB and TPD—21.2 J K^−1^ mol^−1^). This allows the use of the temperature dependencies of the methyl group contributions obtained in refs. [18,20] for an accurate estimation of the heat capacity of *m*-MTDAB in the ideal gas state.

All calculated parameters, additional details on the computation procedure, and the ideal gas phase heat capacities of mCP, TCB, TDAB, and *m*-MTDAB between 200 and 600 K are available in the Supplementary Materials (Tables S13–S18, Figures S4–S6).

## 5. Conclusions

In this study, we have determined the thermodynamic parameters of phase transitions for four organic semiconductors: mCP, TCB, TDAB, and *m*-MTDAB. Using several independent experimental techniques—including differential scanning calorimetry, fast scanning calorimetry, and solution calorimetry—combined with computational methods of quantum chemistry and statistical thermodynamics, we have obtained a self-consistent set of thermodynamic parameters for melting, vaporization, sublimation, and polymorph transition of the studied compounds in a wide temperature range.

The integration of these complementary methods is essential, as for compounds with high molecular weights and high melting points—such as the compounds studied in this work—the absolute errors in heat capacity measurements accumulate significantly upon extrapolation to lower temperatures. In this context, solution calorimetry proves particularly valuable alongside more widely used experimental techniques that operate in the high-temperature region, ensuring the reliability of the derived thermodynamic parameters.

A comparative analysis of the obtained results with the available literature data has been performed. Additionally, the additivity of a number of thermodynamic properties for mCP, TCB, TDAB, and *m*-MTDAB has been tested using previously measured parameters for structurally related compounds. The study confirms the practical utility of the derived data in predicting properties for compounds with similar molecular structures.

## Figures and Tables

**Table 1:** Melting properties of studied compounds.

Compound	*T*_m_/K	$\Delta_{cr}^{l}H(T_{m})$ /kJ mol^−1^	Ref.
mCP	450.84 ± 0.08 ^a^	31.5 ± 0.1 ^a^	[17]
	451.75	–	[33]
	453.8 ± 0.2 ^a^	27.1 ± 1.4 ^a^	[16]
	458.15	–	[34]
	460.15	–	[35]
	449.3 ± 1.0 ^b^	27.5 ± 1.2 ^b^	This work
TCB	467.15	–	[27]
	547.15	33.7	[26]
	549.15	38	[28]
	544.9 ± 1.2 ^b^	32.0 ± 0.5 ^b^	This work, polymorph B
	570.15	–	[29]
	565.6 ± 1.1	28.2 ± 0.3 ^b^	This work, polymorph C
	597.5 ± 1.2 ^a^	41.7 ± 2.5 ^a^	[16]
	599.15	63	[28]
	601.15	57.4	[26]
	599.2 ± 1.2 ^b^	56.3 ± 0.8 ^b^	This work, polymorph A
TDAB	526.3 ± 0.1 ^a^	62.1 ± 0.8 ^a^	[15]
	530.15	–	[36]
	530.4	–	[37]
	527.8 ± 1.0 ^b^	57.9 ± 0.6 ^b^	This work
*m*-MTDAB	455.8 ± 0.1 ^a^	50.3 ± 0.5 ^a^	[15]
	456.15	–	[36]
	457.1 ± 1.4 ^b^	48.8 ± 0.9 ^b^	This work

^a^ Uncertainty reported by the authors; ^b^ expanded uncertainty *U* (0.95 level of confidence, k ≈ 2), including the reproducibility of the measurement and calibration (see Section S3 of the Supplementary Materials).

**Table 2:** Comparison of selected structural parameters and isobaric heat capacities at 298.15 K for mCP, TCB, TDAB and *m*-MTDAB calculated in this work and in ref. [15] ^a^.

Property	This Work	Costa et al.
mCP		
Total energy/hartree	−1264.863	–
r(C_b_–N)/Å	1.421	–
*φ*(C_b_–N–C_carb_)/degree	119.8	–
*τ*(C_b_–C_b_–N–C_carb_)/degree	121.8	–
*C*_p/m_(g, 298.15 K)/J K^−1^ mol^−1^	415.5	–
TCB		
Total energy/hartree	−1781.353	–
r(C_b_–N)/Å	1.414	–
*φ*(C_b_–N–C_carb_)/degree	119.7	–
*τ*(C_b_–C_b_–N–C_carb_)/degree	125.9	–
*C*_p/m_(g, 298.15 K)/J K^−1^ mol^−1^	583.2	–
TDAB		
Total energy/hartree	−1784.911	−1784.704
r(C_b_–N)/Å	1.399	1.422
r(N–C_Ph_)/Å	1.400	1.422
*φ*(C_b_–N–C_Ph_)/degree	120.0	120.1
*φ*(C_Ph_–N–C_Ph_)/degree	120.2	119.8
*τ*(C_b_–C_b_–N–C_Ph_)/degree	145.0	136.8
*τ*(C_b_–N–C_Ph_–C_Ph_)/degree	37.7	42.8
*C*_p/m_(g, 298.15 K)/J K^−1^ mol^−1^	625.1	642.8
*m*-MTDAB		
Total energy/hartree	−1902.890	−1902.547
r(C_b_–N)/Å	1.414	1.420
r(N–C_Ph_)/Å	1.429	1.421
r(N–C_PhCH3_)/Å	1.424	1.423
*φ*(C_b_–N–C_Ph_)/degree	120.0	120.2
*φ*(C_b_–N–C_PhCH3_)/degree	121.4	120.1
*φ*(C_Ph_–N–C_Ph_)/degree	118.6	119.7
*τ*(C_b_–C_b_–N–C_Ph_)/degree	147.3	141.2
*τ*(C_b_–C_b_–N–C_PhCH3_)/degree	149.7	139.2
*τ*(C_b_–N–C_Ph_–C_Ph_)/degree	55.0	41.3
*τ*(C_b_–N–C_PhCH3_–C_PhCH3_)/degree	42.3	42.8
*C*_p/m_(g, 298.15 K)/J K^−1^ mol^−1^	689.3	706.7

^a^ The notations are the following: C_b_ denotes the carbon atom of the central benzene ring, C_carb_—carbon atom of the carbazolyl fragment, C_Ph_—carbon atom of the phenyl group, and C_PhCH3_—carbon atom of the *m*-tolyl group.

**Table 3:** Coefficients of Equation (1).

Compound	Phase	Method	Temperature Range/K	*a*	*b*	*c*·10^3^	RSME (SD) ^a^/ J K^−1^ mol^−1^
mCP	liquid	DSC	350–512	284.5	1.050	0	1.2
	crystal	DSC	281–380	−19.9	1.561	0	1.1
TCB	liquid	FSC	455–525	271.9	1.768	0	0.4
	crystal A	DSC	277–500	−20.0	2.259	−0.403	2.7
TDAB	liquid	FSC	437–492	−1094.3	8.320	−7.392	0.5
	crystal	DSC	278–472	−117.7	2.984	−1.061	2.3
*m*-MTDAB	liquid	FSC	367–416	453.6	1.726	0	0.6
	crystal	DSC	278–432	−45.2	2.609	0	3.2

^a^ Root mean square error—$RSME=\sqrt{{\sum \left({\hat{y}}_{i}-y_{i}\right)}^{2}/n}$; ${\hat{y}}_{i}$—fitted value, $y_{i}$—experimental value, *n*—number of values.

**Table 4:** Experimental and literature heat capacities of crystalline mCP, TDAB, and *m*-MTDAB.

Compound	*C*_p,m_(cr, exp)/ J K^−1^ mol^−1^	*C*_p,m_(cr, lit)/ J K^−1^ mol^−1^	Ref.
mCP	446	445.2	[17]
TDAB	678	655.6	[15]
*m*-MTDAB	733	738.8	[15]

**Table 5:** Parameters of Clarke–Glew equation (Equation (2)).

Compound	Phase	$ln(p\;(T_{0})/Pa)$	$\Delta_{cr/l}^{g}H(T_{0})$ /kJ mol^−1^	$\Delta_{cr/l}^{g}C_{p,m}$ /J K^−1^ mol^−1^
mCP	liquid ^a^	−23.827	158.7	−152
	crystal ^a^	−25.748	164.8	−40
TCB	liquid ^a^	−38.896	220.4	−207
	crystal A ^b^	−43.512	226.4	−62
TDAB	liquid ^a^	−29.927	206.3	−320
	crystal ^a^	−35.879	223.3	−138
*m*-MTDAB	liquid ^a^	−31.506	213.1	−324
	crystal ^b^	−35.829	231.6	−143

^a^ Fit of the experimental values; ^b^ calculated using sublimation and melting data.

**Table 6:** Solution and fusion enthalpies of mCP, TCB, TDAB, and *m*-MTDAB at *T*_0_ = 298.15 K.

Compound	$\Delta_{soln}H^{A/C_{6}H_{6}}(cr,T_{0})$ /kJ mol^−1^	$\Delta_{cr}^{l}H(T_{0})$ (Equation (5)) /kJ mol^−1^	$\Delta_{cr}^{l}H(T_{0})$ (Equation (4)) /kJ mol^−1^	$\Delta_{cr}^{l}H(T_{0})$ (lit) /kJ mol^−1^	Ref.
mCP	14.3 ± 0.2 ^a^	13.3 ± 1.0 ^b^	10.5 ± 4.0 ^c^	19.4 ± 2.1	[16]
TCB	15.7 ± 0.2 ^a^	14.7 ± 1.0 ^b^	10.2 ± 8.1 ^c^	26.8 ± 3.9	[16]
TDAB	21.1 ± 0.5 ^a^	20.1 ± 1.1 ^b^	19.6 ± 7.5 ^c^	50.7 ± 2.3	[15]
*m*-MTDAB	27.0 ± 0.4 ^a^	26.0 ± 1.1 ^b^	22.5 ± 6.6 ^c^	42.4 ± 1.6	[15]

^a^ Combined uncertainty of solution enthalpy measurements including reproducibility and calibration uncertainties; ^b^ combined uncertainty of solution enthalpies of hypothetical liquid and crystalline compounds; ^c^ combined uncertainty of the fusion enthalpy at *T*_m_ and temperature correction.

**Table 7:** The experimental and calculated heat capacities of crystalline organic semiconductors at 298.15 K.

Compound	Phase	*C*_p,m_(Exp.) /J K^−1^ mol^−1^	*C*_p,m_(Calc.) /J K^−1^ mol^−1^	*C*_p,m_(Calc., Ref. [40]) /J K^−1^ mol^−1^	*C*_p,m_(Calc., Ref. [41]) /J K^−1^ mol^−1^
mCP	liquid	598	–	575.4	624.6
	crystal	446	–	450.8	439.2
CBP	liquid	748	–	686.6	742.4
	crystal	542	–	535.8	526.2
TCB	liquid	799	839	797.1	871.5
	crystal	619 ^a^	624	622.3	606.3
DDP	liquid	669	–	625.1	674.4
	crystal	463	–	490.5	546.8
TPB	liquid	797	–	736.3	792.2
	crystal	553	–	575.4	633.8
TDAB	liquid	933	929	871.7	946.2
	crystal	678	648	681.8	767.7
*p*-TTP	liquid	684	–	678.3	731.2
	crystal	502	–	541.9	602.0
TPD	liquid	814	–	789.5	849.0
	crystal	596	–	626.9	689.0
*m*-MTDAB	liquid	968	954	951.5	1031.4
	crystal	733	713	759.0	850.5

^a^ Polymorph A.

**Table 8:** Literature and experimental sublimation (S) and vaporization (V) enthalpies of mCP, TCB, TDAB, and *m*-MTDAB.

Compound	Transition	*T*/K	$\Delta_{cr/l}^{g}H(T)$ / J K^−1^ mol^−1^	$\Delta_{cr/l}^{g}H(T)$ (Equation (4))/ J K^−1^ mol^−1 a,b^	$\Delta_{cr/l}^{g}H(T_{0})$ / J K^−1^ mol^−1 b,c,d^	Ref.
mCP	V	600	118.6 ± 0.4	114.4 ± 4.5	-	[17]
		450.84 ± 0.08	128.2 ± 0.8 ^d^	135.0 ± 3.2	-	[17]
		466	135.2 ± 0.5	132.9 ± 3.1	147.1 ± 1.8	[16]
		482.0	130.7 ± 3.1 ^e^	-	158.7 ± 5.1	This work
	S	450.84 ± 0.08	159.6 ± 0.8 ^f^	160.7 ± 4.2	164.5 ± 1.5	[17]
		-	-	-	166.5 ± 2.7 ^f^	[16]
		430.7	159.5 ± 4.2 ^e^	-	164.8 ± 4.8	This work
TCB	V	-	-	-	178.5 ± 4.8 ^f^	[16]
		528.5	172.7 ± 3.3 ^e^	-	220.3 ± 12.3	This work
	S ^f^	569.2	199.9 ± 0.4	208.0 ± 5.8	205.3 ± 2.7	[16]
		599.2	211.8 ± 5.7 ^b,g^	-	230.5 ± 10.7 ^f^	This work
TDAB	V	-	-	-	150.1 ± 3.0 ^f^	[15]
		492.2	144.2 ± 3.2 ^e^	-	193.2 ± 11.1	This work
	S	493.37	198.3 ± 0.4	196.7 ± 4.0	200.8 ± 2.0	[15]
		480.9	198.1 ± 4.0 ^e^	-	211.0 ± 6.6	This work
*m*-MTDAB	V	482.0	153.6 ± 3.4 ^e^	-	197.3 ± 11.2	This work
	S	457.1	207.8 ± 3.9 ^b,f^	-	219.8 ± 6.3 ^f^	This work

^a^ The values calculated at *T* (3rd column) using our experimental data; ^b^ the combination of enthalpy and temperature correction uncertainties; ^c^ *T*_0_ = 298.15 K; ^d^ calculated using Kirchoff’s Law of Thermochemistry; ^e^ the standard uncertainties were evaluated as previously [43,44]; ^f^ sublimation of crystal A; ^g^ calculated using Hess’s law.

## Data Availability

All data are available on request from the corresponding author.

## Supplementary Materials

The following supporting information can be downloaded at: https://www.mdpi.com/article/10.3390/molecules31091435/s1, Figure S1: XRPD spectra of TCB polymorphs. Figure S2: Heat flow curves of crystalline (dashed lines) and liquid (solid lines, also include glass) indomethacin on the surface of UFS1 chip sensor. Heating and cooling at ±500 K s^−1^ are shown in red and blue, respectively. Frame represents interval where heat capacities were treated. Figure S3: Deviation plots of measured vapor pressures from the recommended literature values for phenanthrene (left) and methyl octadecenoate (right). Figure S4: Potential energy surface for internal rotation in mCP. A dihedral angle of 0 degrees corresponds to the optimal configuration. Figure S5: Potential energy surface for internal rotation in TCB. A dihedral angle of 0 degrees corresponds to the optimal configuration. Figure S6: Potential energy surfaces for internal rotations in TDAB. A dihedral angle of 0 degrees corresponds to the optimal configuration. Figure S7: Chemical structures of previously studied DDP (*N*,*N*,*N*′,*N*′-tetraphenyl-*p*-phenylenediamine), *p*-TTP (*N*,*N*′-diphenyl-*N*,*N*′-di-*p*-tolylbenzene), TPB (*N*,*N*,*N*′,*N*′-tetraphenylbenzidine), CBP (4,4′-bis(*N*-carbazolyl)-1,1′-biphenyl), and TPD (4,4′-bis(*m*-tolylphenylamino)biphenyl). Table S1: The origin and purity of studied and auxiliary compounds used in this work. Table S2: Unsmoothed experimental values of molar heat capacities of crystalline anthracene, thioxanthone, and indium used for validation of the performance of the heat capacity measurements. Experimental pressure is 0.1 MPa. Table S3: Fusion enthalpies and melting points of mCP, TCB, TDAB and m-MTDAB measured in this work at 0.1 MPa. Table S4: Isobaric heat capacities of mCP, TCB, TDAB and m-MTDAB measured in this work at 0.1 MPa. Table S5: Isobaric heat capacities of supercooled liquid *o*-terphenyl and indomethacin measured in this work using FSC at 0.1 MPa. Table S6: Isobaric heat capacities of supercooled liquid phases of TCB, TDAB and m-MTDAB measured in this work at 0.1 MPa. Table S7: Unsmoothed values of saturated vapor pressures of liquid phenanthrene and methyl octadecenoate and their comparison with recommended literature values. Table S8: Values of saturated vapor pressures of mCP, TCB, TDAB, and m-MTDAB measured in this work using thermogravimetry–fast scanning calorimetry. Table S9: Vapor pressures above crystalline phases of TCB and m-MTDAB calculated on the base of the vaporization and melting data. Table S10: Parameters of Clarke–Glew equation (Equation (3)) used for vapor pressure calculations above crystalline phases of TCB and m-MTDAB. Table S11: Experimental enthalpies of solution of propanol-1 and potassium chloride in water measured at 298.15 K and 0.1 MPa. Table S12: Experimental solution enthalpies of mCP, TCB, TDAB and m-MTDAB in benzene measured in this work at 298.15 K and 0.1 MPa. Table S13: Cartesian coordinates of mCP, TCB and TDAB optimized with B3LYP/6-31+G(d,p). Table S14: Reduced moments of inertia *I*_r_ of the rotating tops of mCP, TCB and TDAB. Table S15: Computed fundamental vibrational wavenumbers of mCP, TCB and TDAB used in the calculation of the ideal gas heat capacities. The frequencies corresponding to the internal rotation were identified according to Ayala and excluded from the table and further calculations. Table S16: Energy levels of hindered rotors encountered in mCP, TCB and TDAB (up to a frequency of 3000 cm^−1^). Table S17: Contributions of internal rotation to the heat capacity of mCP, TCB and TDAB. Table S18: Contributions of vibration and internal rotation to the heat capacities of mCP, TCB and TDAB, as well as isochoric and isobaric heat capacities calculated in this work. Table S19: Ideal gas heat capacities at 298.15 K for organic semiconductors and the corresponding group contributions calculated from these compounds. Table S20: Group contributions to liquid and crystal heat capacities at 298.15 K. Equations (S1)–(S8): Details of FSC calibration and details of the computations. The following references are additionally cited in the Supplementary Materials: [53,54,55,56,57,58,59,60,61,62,63,64,65,66,67,68,69,70,71,72,73].

## Abbreviations

The following abbreviations are used in this manuscript:

DSC	Differential scanning calorimetry
FSC	Fast scanning calorimetry
SC	Solution calorimetry
HPLC	High performance liquid chromatography
mCP	1,3-bis(9H-carbazol-9-yl)benzene
CBP	4,4′-bis(*N*-carbazolyl)-1,1′-biphenyl
TCB	1,3,5-tri(9H-carbazol-9-yl)benzene
DDP	*N*,*N*,*N*′,*N*′-tetraphenyl-*p*-phenylenediamine
TPB	*N*,*N*,*N*′,*N*′-tetraphenylbenzidine
TDAB	1,3,5-tris(diphenylamino)benzene
*p*-TTP	*N*,*N*′-diphenyl-*N*,*N*′-di-*p*-tolylbenzene
TPD	4,4′-bis(*m*-tolylphenylamino)biphenyl
*m*-MTDAB	1,3,5-tris[(3-methylphenyl)phenylamino]benzene
