# Supplementary material for: Phase Transition Thermodynamics of Organic Semiconductors 1,3-Bis(9H-carbazol-9-yl)benzene, 1,3,5-Tri(9H-carbazol-9-yl)benzene, 1,3,5-Tris(diphenylamino)benzene, and 1,3,5-Tris[(3-methylphenyl)phenylamino]benzene
Source: Molecules. 2026 Apr 26;31(9):1435. doi: 10.3390/molecules31091435 (PMC13165118; doi:10.3390/molecules31091435)
Supplement: Supplementary file 1 [file molecules-31-01435-s001.zip › molecules-4188038-supplementary.pdf]

Supplementary Material

Phase transition thermodynamics of organic semiconductors 1,3-bis(9H-carbazol-9-yl)benzene, 1,3,5-tri(9H-carbazol-9-yl)benzene, 1,3,5-tris(diphenylamino)benzene, 1,3,5-tris[(3-methylphenyl)phenylamino]benzene

Airat A. Notfullin[1], Dmitrii N. Bolmatenkov, Andrey A. Sokolov, Ilya S. Balakhontsev, Mansur B. Khisamiev, Boris N. Solomonov[2], and Mikhail I. Yagofarov

Department of Physical Chemistry, Kazan Federal University, Kremlevskaya str. 18, 420008 Kazan, Russia

1 Materials

Table S1
The origin and purity of studied and auxiliary compounds used in this work.

Compound	CAS	Supplier	Purification method	Purity
mCP	550378-78-4	Hotspot Biotechnology	sublimation	0.98[a]/0.998[b]
TCB	148044-07-9	Hotspot Biotechnology	sublimation	0.98[a]/0.992[b]
TDAB	126717-23-5	Hotspot Biotechnology	sublimation	0.98[a]/0.991[b]
m-MTDAB	138143-23-4	Hotspot Biotechnology	sublimation	0.98[a]/0.994[b]
benzene	71-43-2	Ekos-1	distillation	0.99[a]/0.999[c]
acetonitrile	75-05-8	Merck	–	0.9999[a]
toluene	108-88-3	Chimmed	distillation	0.99[a]/0.999[d]
indium	7440-74-6	PerkinElmer	–	0.99999[a]
zinc	7440-66-6	PerkinElmer	–	0.99999[a]
anthracene	120-12-7	Aldrich	–	0.99[a]/0.999[d]
thioxanthone	492-22-8	TCI	–	0.999[a]
sapphire[e]	1344-28-1	Perkin Elmer	–	not provided
biphenyl	92-52-4	NETZSCH	–	0.995[a]/0.999[d]
benzoic acid	65-85-0	NETZSCH	–	0.995[a]/0.999[d]
N-methylbenzamide	613-93-4	TCI	dried *in vacuo*	0.99[a]/0.999[d]
phenanthrene	85-01-8	Aldrich	–	0.98[a]/0.998[d]
o-terphenyl	84-15-1	Aldrich	–	0.99[a]/0.999[d]
indomethacin	53-86-1	Aldrich	–	0.985[a]/0.9954[b]
methyl octadecenoate	112-61-8	Aldrich	–	0.99[a]/0.998[d]

[a] Mass fraction, stated by the supplier;
[b] Mole fraction, found using HPLC;
[c] Mass fraction determined by Karl Fisher titration;

[1] *Correspondence:* notfullinair@gmail.com;
[2] *Correspondence:* boris.solomonov@kpfu.ru.

[d] Mass fraction purity determined using gas chromatography (7890 B gas chromatograph (Agilent, Santa Clara, CA, USA));
[e] Sapphire disc provided by Perkin Elmer (USA) as a reference material for the heat capacity measurements.

2 X-ray powder diffraction analysis

XRPD spectra for TCB were recorded on a MiniFlex 600 diffractometer (Rigaku) equipped with a D/teX Ultra detector using Cu Kα radiation (40 kV, 15 mA). Measurements were taken at room temperature from 3° to 50° 2θ with a step size of 0.02° and a counting time of 0.24 s per point, without sample rotation. The spectra for forms A, B, and C of TCB are provided in Figure S1.

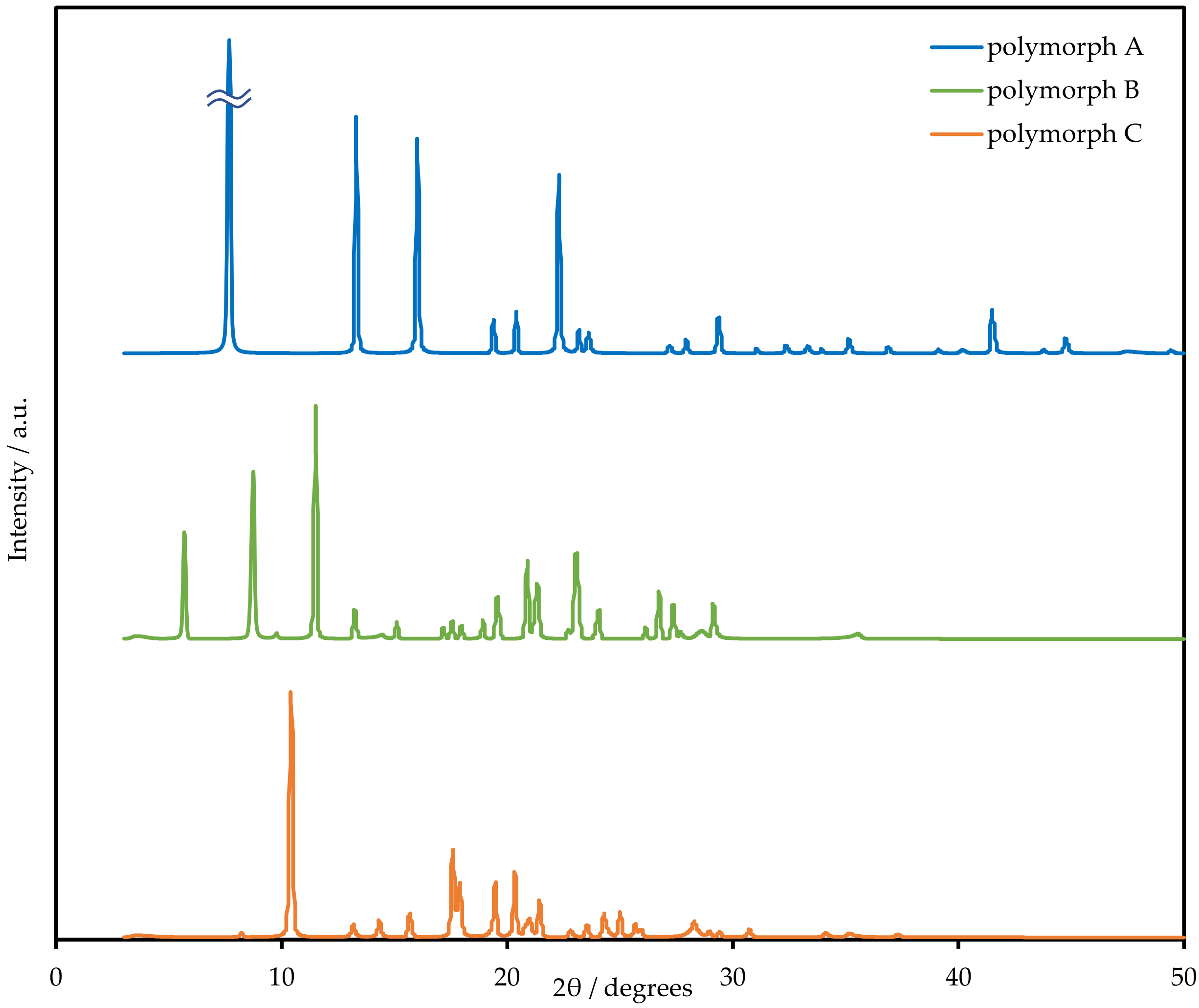

Figure S1. XRPD spectra of TCB polymorphs.

3 Differential scanning calorimetry

DSC was calibrated according to the manufacturer's recommendation using indium and zinc samples provided by manufacturer. Before the measurements, aluminum crucibles were conditioned at 423 K for 5 minutes to eliminate impurities. Each value (onset temperature and area of the peak) was determined three times. The reproducibilities of heat flow and temperature calibration (0.95 level of confidence, coverage factor 2) were equal to 1 % and 0.1 K, respectively.

The correctness of determination of the heat capacity was checked by measuring the heat capacity of crystalline anthracene, thioxanthone and indium. The heat capacities were measured using three-step procedure. Program including isotherm (3 min), heating with a rate of 10 K min^{-1} and

second isotherm (3 min) was repeated for empty crucible, standard sapphire disc (m = 27.96 mg) and sample with a mass of 5-15 mg.

The data were treated using Pyris Software (Version 13.4) with built-in standard values for sapphire heat capacities, indium and zinc melting points, and indium fusion enthalpy.

The measured heat capacities were compared with the recommended values for anthracene (Table S2) [1] and indium [2] and with our previous adiabatic calorimetry data for thioxanthone [3].

To check the possible influence the sample mass on the measured values according to Ref. [4], we varied the mass of anthracene samples from 3.52 mg 10.80 mg. No systematic influence was observed.

Relative deviation between measured and reference values varied from -1.5 % to 1.7 %.

Table S2
Unsmoothed experimental values of molar heat capacities of crystalline anthracene, thioxanthone, and indium used for validation of the performance of the heat capacity measurements. Experimental pressure is 0.1 MPa.

T / K	$C_{p,m}$ (exp) / J mol^{-1} K^{-1}	$U(C_{p,m})^a$ / J mol^{-1} K^{-1}	$C_{p,m}$ (lit)^b / J mol^{-1} K^{-1}	Relative deviation / %
		anthracene (cr)/ m = 3.52 mg		
280.0	196.9	5.9	196.0	0.5
290.0	205.2	6.2	204.0	0.6
300.0	210.4	6.3	211.9	-0.7
310.0	220.1	6.6	219.8	0.1
320.0	228.4	6.9	227.6	0.3
330.0	236.6	7.1	235.3	0.6
340.0	244.0	7.3	243.0	0.4
350.0	251.8	7.6	250.6	0.5
360.0	259.0	7.8	258.2	0.3
370.0	266.3	8.0	265.7	0.2
380.0	273.8	8.2	273.1	0.2
390.0	281.6	8.4	280.5	0.4
400.0	288.8	8.7	287.8	0.4
		anthracene (cr)/ m = 6.86 mg		
280.0	194.2	5.8	196.0	-0.9
290.0	202.0	6.1	204.0	-1.0
300.0	209.9	6.3	211.9	-1.0
310.0	217.8	6.5	219.8	-0.9
320.0	226.1	6.8	227.6	-0.7
330.0	233.4	7.0	235.3	-0.8
340.0	240.3	7.2	243.0	-1.1
350.0	247.3	7.4	250.6	-1.3
360.0	254.3	7.6	258.2	-1.5
370.0	262.4	7.9	265.7	-1.3
380.0	270.4	8.1	273.1	-1.0
390.0	278.4	8.4	280.5	-0.8
400.0	287.0	8.6	287.8	-0.3
		anthracene (cr)/ m = 10.80 mg		
280.0	194.5	5.9	196.0	-0.7
290.0	202.2	6.1	204.0	-0.9

300.0	211.0	6.4	211.9	-0.4
310.0	218.7	6.6	219.8	-0.5
320.0	226.9	6.8	227.6	-0.3
330.0	234.5	7.1	235.3	-0.4
340.0	242.0	7.3	243.0	-0.4
350.0	250.1	7.5	250.6	-0.2
360.0	257.2	7.7	258.2	-0.4
370.0	265.0	8.0	265.7	-0.2
380.0	272.5	8.2	273.1	-0.2
390.0	280.4	8.4	280.5	0.0
400.0	287.9	8.6	287.8	0.0
thioxanthone (cr)				
280.0	209.3	6.3	209.9	-0.3
290.0	214.1	6.5	217.0	-1.4
300.0	221.6	6.7	224.1	-1.1
310.0	229.4	6.9	231.1	-0.7
320.0	237.2	7.1	238.1	-0.4
330.0	242.2	7.3	245.0	-1.1
340.0	248.8	7.6	251.9	-1.2
350.0	255.7	7.8	258.7	-1.1
360.0	263.1	8.0	265.4	-0.9
370.0	271.1	8.2	272.1	-0.4
380.0	280.5	8.4	278.7	0.6
390.0	289.9	8.6	285.3	1.6
400.0	296.9	8.8	291.9	1.7
indium (cr)				
300.0	27.0	0.8	26.9	0.2
310.0	27.1	0.8	27.1	-0.1
320.0	27.4	0.8	27.3	0.3
330.0	27.5	0.8	27.5	-0.1
340.0	27.6	0.8	27.7	-0.4
350.0	27.9	0.8	27.9	0.1
360.0	28.1	0.8	28.1	0.3
370.0	28.4	0.8	28.3	0.4
380.0	28.6	0.9	28.5	0.6
390.0	28.8	0.9	28.7	0.7
400.0	29.0	0.9	28.9	0.5

[a] The uncertainties correspond to expanded uncertainty (U) at a level of confidence of 95%, and coverage factor of 2. $u(p)$ = 5 kPa, $u(T)$ in measurements by DSC is 0.1 K where u is standard uncertainty;

[b] Reference data were taken from Ref. [1] for anthracene, from Ref. [3] for thioxanthone, and from Ref. [2] for indium and smoothed by second-order polynomial.

Table S3

Fusion enthalpies and melting points of mCP, TCB, TDAB and m-MTDAB measured in this work at 0.1 MPa [a].

Compound	mass / mg	T_m / K	$\Delta_{cr}^{l}H(T_m)$/ kJ mol^{-1}
mCP	7.99	449.39	26.35
	5.02	449.46	27.78
	5.82	449.08	28.39
	**Average**	**449.3±1.0**[b]	**27.5±1.2**[b]
TCB, crystal A	1.58	598.62	56.22
	2.48	598.68	57.37
	2.73	599.84	56.22

	3.07	600.14	56.35
	2.99	598.90	55.49
	**Average**	**599.2±1.2[b]**	**56.3±0.8[b]**
TCB, crystal B	2.44	544.03	31.64
	2.73	544.18	31.47
	2.99	545.16	32.43
	1.02	545.46	32.25
	3.52	545.47	32.36
	**Average**	**544.9±1.2[b]**	**32.0±0.5[b]**
TCB, crystal C	2.44	565.73	28.11
	3.61	566.01	27.81
	6.72	566.17	28.18
	3.91	565.01	28.39
	3.07	564.91	28.32
	**Average**	**565.6±1.1[b]**	**28.2±0.3[b]**
TDAB	5.20	527.60	57.78
	3.26	527.98	57.82
	4.62	527.82	58.16
	**Average**	**527.8±1.0[b]**	**57.9±0.6[b]**
m-MTDAB	5.03	456.58	48.23
	4.20	456.71	48.71
	5.08	458.03	49.51
	**Average**	**457.1±1.4[b]**	**48.8±0.9[b]**

[a] Standard uncertainty $u(p)$ = 5 kPa;

[b] Expanded uncertainty U (0.95 level of confidence, k ≈ 2), including the reproducibility of the measurement and calibration.

Table S4

Isobaric heat capacities of mCP, TCB, TDAB and m-MTDAB measured in this work at 0.1 MPa [a].

***T* / K**	**$C_{p,m}$ / J mol^{-1} K^{-1}**	**$U(C_{p,m})$ [b] / J mol^{-1} K^{-1}**	***T* / K**	**$C_{p,m}$ / J mol^{-1} K^{-1}**	**$U(C_{p,m})$ [b] / J mol^{-1} K^{-1}**
	mCP, liquid			TCB, crystal A	
350.0 [c]	652.8	19.6	277.0	576.0	17.3
355.0 [c]	657.3	19.7	280.0	581.7	17.5
360.0 [c]	661.4	19.8	285.0	590.9	17.7
365.0 [c]	666.7	20.0	290.0	601.8	18.1
370.0 [c]	672.8	20.2	295.0	611.3	18.3
375.0 [c]	678.1	20.3	300.0	621.0	18.6
380.0 [c]	683.4	20.5	305.0	631.9	19.0
385.0 [c]	687.5	20.6	310.0	643.1	19.3
390.0 [c]	692.8	20.8	315.0	653.4	19.6
395.0 [c]	699.8	21.0	320.0	664.3	19.9
400.0 [c]	704.7	21.1	325.0	675.5	20.3
405.0 [c]	710.0	21.3	330.0	686.1	20.6
410.0 [c]	716.1	21.5	335.0	690.5	20.7
415.0 [c]	721.4	21.6	340.0	695.1	20.9
420.0 [c]	726.7	21.8	345.0	705.3	21.2
425.0 [c]	733.3	22.0	350.0	715.8	21.5
430.0 [c]	735.8	22.1	355.0	726.3	21.8
435.0 [c]	741.1	22.2	360.0	737.6	22.1
440.0 [c]	745.9	22.4	365.0	748.3	22.4
445.0 [c]	751.6	22.5	370.0	757.9	22.7
450.0	754.5	22.6	375.0	769.5	23.1
455.0	759.4	22.8	380.0	780.2	23.4

460.0	765.1	23.0
465.0	772.1	23.2
470.0	780.6	23.4
475.0	783.0	23.5
480.0	789.4	23.7
485.0	794.7	23.8
490.0	800.1	24.0
495.0	803.7	24.1
500.0	809.2	24.3
505.0	812.9	24.4
510.0	820.1	24.6
512.0	822.1	24.7
	mCP, crystal	
281.0	418.3	12.5
285.0	424.6	12.7
290.0	433.4	13.0
295.0	441.0	13.2
300.0	448.9	13.5
305.0	456.7	13.7
310.0	463.9	13.9
315.0	472.2	14.2
320.0	480.4	14.4
325.0	488.2	14.6
330.0	495.7	14.9
335.0	503.9	15.1
340.0	511.2	15.3
345.0	519.3	15.6
350.0	527.1	15.8
355.0	535.0	16.0
360.0	544.5	16.3
365.0	552.1	16.6
370.0	559.0	16.8
375.0	565.2	17.0
380.0	569.9	17.1
	m-MTDAB, crystal	
278.0	680.9	20.4
280.0	684.0	20.5
285.0	697.1	20.9
290.0	708.9	21.3
295.0	721.6	21.6
300.0	733.8	22.0
305.0	749.0	22.5
310.0	762.0	22.9
315.0	776.7	23.3
320.0	791.0	23.7
325.0	805.7	24.2
330.0	818.9	24.6
335.0	832.9	25.0
340.0	846.6	25.4
345.0	858.4	25.8
350.0	872.7	26.2
355.0	883.6	26.5
360.0	894.8	26.8
385.0	790.2	23.7
390.0	800.7	24.0
395.0	811.7	24.4
400.0	823.3	24.7
405.0	832.6	25.0
410.0	842.2	25.3
415.0	852.2	25.6
420.0	860.9	25.8
425.0	869.1	26.1
430.0	878.5	26.4
435.0	886.0	26.6
440.0	895.9	26.9
445.0	905.5	27.2
450.0	916.4	27.5
455.0	923.9	27.7
460.0	932.1	28.0
465.0	940.3	28.2
470.0	952.1	28.6
475.0	959.8	28.8
480.0	971.8	29.2
485.0	982.6	29.5
490.0	990.8	29.7
495.0	999.7	30.0
500.0	1006.6	30.2
	TDAB, crystal	
278.0	629.0	18.9
280.0	633.4	19.0
285.0	645.8	19.4
290.0	657.7	19.7
295.0	668.4	20.1
300.0	680.6	20.4
305.0	693.4	20.8
310.0	705.0	21.1
315.0	717.1	21.5
320.0	727.3	21.8
325.0	740.6	22.2
330.0	753.4	22.6
335.0	765.8	23.0
340.0	778.6	23.4
345.0	790.2	23.7
350.0	796.0	23.9
355.0	807.2	24.2
360.0	819.4	24.6
365.0	830.0	24.9
370.0	840.4	25.2
375.0	852.5	25.6
380.0	863.0	25.9
385.0	876.0	26.3
390.0	884.5	26.5
395.0	900.0	27.0
400.0	905.0	27.1
405.0	913.1	27.4
410.0	925.2	27.8

365.0	907.2	27.2	415.0	936.3	28.1
370.0	919.1	27.6	420.0	947.1	28.4
375.0	930.4	27.9	425.0	955.9	28.7
380.0	940.8	28.2	430.0	963.9	28.9
385.0	954.5	28.6	435.0	975.9	29.3
390.0	971.0	29.1	440.0	987.9	29.6
395.0	985.6	29.6	445.0	998.5	30.0
400.0	997.4	29.9	450.0	1008.2	30.2
405.0	1007.4	30.2	455.0	1023.8	30.7
410.0	1019.5	30.6	460.0	1031.3	30.9
415.0	1033.5	31.0	465.0	1042.2	31.3
420.0	1047.8	31.4	470.0	1052.8	31.6
425.0	1063.3	31.9	472.0	1057.6	31.7
430.0	1081.7	32.4			
432.0	1089.1	32.7			

[a] Standard uncertainty *u(p)* = 5 kPa;

[b] Expanded uncertainty *U* (0.95 level of confidence, k ≈ 2) of the heat capacity, including the reproducibility of the measurement and calibration;

[c] Supercooled liquid phase.

The prediction uncertainty $U(T)$ of extrapolated heat capacities at the temperatures outside the measurement range was calculated as follows [5]:

$$U(T) = U_{\exp}\sqrt{1+\frac{1}{n-1}+\frac{(T-T_{\mathrm{av}})^2}{\sum (T_{\mathrm{i}}-T_{\mathrm{av}})^2}} \tag{S1}$$

where n is a number of degrees of freedom for the set of points; T_{av} is the mean arithmetic of temperatures at which single $C_{\mathrm{p,m}}(T)$ values were obtained. The uncertainty $U_{\exp}$ was calculated from $C_{\mathrm{p,m}}(T)$ in the middle of the measurement range of temperature using $U_{\exp}$, provided in Table S4.

In Table S4, for the sake of readability, the heat capacity values of the condensed phases are presented with a temperature step of 5 K. In reality, however, all measurements were performed with a step of 0.1 K or less (both for DSC and FSC). Given the wide temperature range of the measurements and the large number of experimental data points, the second and third components under the square root in the equation are negligibly small. As a result, the error values of the linearly extrapolated heat capacities become practically equal to those of the experimental ones.

4 Fast scanning calorimetry

4.1 Calibration procedure

The UFS1 sensor chip was prepared and calibrated by following the previously developed procedures [6]. Biphenyl, benzoic acid and anthracene were used as temperature standards [1, 7]. All compounds initially were premelted to provide a reproducible temperature contact between the sample and the sensor surface. The melting points of the samples were then measured at various heating rates ranging from 100 K s^{-1} to 5000 K s^{-1}. The obtained values were extrapolated to zero heating rate and subsequently used for calibration. The observed shift between the extrapolated and recommended [1, 7] melting temperatures, ΔT, was then fitted linearly as a function of measured temperature to obtain a temperature correction line. The calibration

accuracy was additionally verified by measuring the melting points of *N*-methylbenzamide [8] and phenanthrene [9]. For both compounds, the measured and literature values were within the range of 1 K. Also, to ensure the uncertainty remains the same at temperatures above 400 K, the melting points of compounds studied in this work (Table S3) was used for comparison. An agreement better than 1 K was observed.

The calibration uncertainty was estimated as reproducibility of melting temperature determination of the abovementioned compounds and was found to be within ±1 K at temperatures up to 600 K.

4.2 Procedure of the heat capacity measurements using FSC

Fast scanning calorimetry enables measurement of the heat capacity ratio of two phases, $C_{p,m}(l, T)/C_{p,m}(cr, T)$, and the calculation of $C_{p,m}(l, T)$ from known $C_{p,m}(cr, T)$.

To measure $C_{p,m}(l, T)/C_{p,m}(cr, T)$, the same sample in two phases should be studied. Heat flow at heating or cooling ($HF(T)$, W) is given by Eq. S2:

$$HF_{h/c}(T) = n \cdot C_{\mathrm{p,m}}(T) \cdot \beta_{h/c} + HL_{h/c}(T) \qquad \text{(S2)},$$

where n is the amount of sample (mole) and HL is heat losses.

Using the same scanning rate at heating and cooling ($\beta_h = -\beta_c$) and assuming that $HL_h = HL_c$, one can derive:

$$n \cdot C_{\mathrm{p,m}}(T) = C_{\mathrm{p}}(T) = \frac{HF_c(T) - HF_h(T)}{2\beta} \qquad \text{(S3)},$$

Thus, recording heat flows for two phases at the same heating rate in a temperature range where sample evaporation is negligible (n is constant), one can obtain:

$$\frac{C_{\mathrm{p,m}}(\mathrm{l},T)}{C_{\mathrm{p,m}}(\mathrm{cr},T)} = \frac{HF_c(\mathrm{l},T) - HF_h(\mathrm{l},T)}{HF_c(\mathrm{cr},T) - HF_h(\mathrm{cr},T)} \qquad \text{(S4)},$$

Example of such measurements for indomethacin are shown in Figure S2.

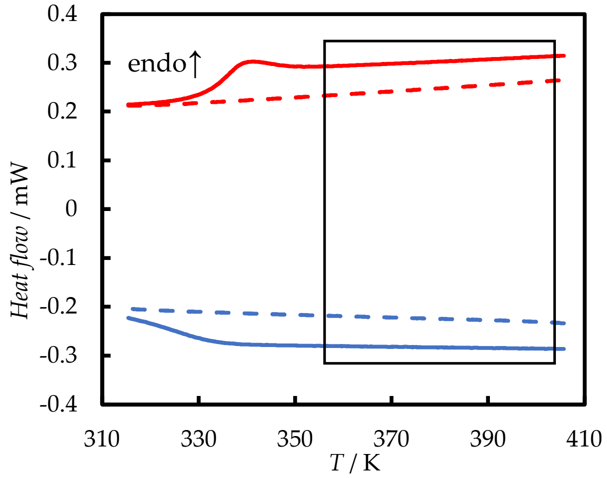

Figure S2. Heat flow curves of crystalline (dashed lines) and liquid (solid lines, also include glass) indomethacin on the surface of UFS1 chip sensor. Heating and cooling at ±500 K s^{-1} are shown in red and blue, respectively. Frame represents interval where heat capacities were treated.

Combined uncertainty of the obtained $C_{p,m}$(l, T) U_r includes uncertainty of $C_{p,m}$(cr, T) (typically 3 %) and reproducibility of the measured $C_{p,m}$(l, T)/$C_{p,m}$(cr, T) ratio (up to 4 %) and equals 5 % in total.

The reliability of such approach was verified using *o*-terphenyl and indomethacin. For *o*-terphenyl, heat capacities of crystalline and supercooled liquid compound measured using adiabatic calorimetry were available from Ref. [10]. For indomethacin, these data were obtained using power-compensated DSC [11]. Five samples of each compound were studied, and obtained $C_{p,m}$(l, T)/$C_{p,m}$(cr, T) were averaged. Within the experimental series, an agreement within 3 % was observed.

Table S5
Isobaric heat capacities of supercooled liquid *o*-terphenyl and indomethacin measured in this work using FSC at 0.1 MPa[a].

T / K	$C_{p,m}$(cr) / J mol^{-1} K^{-1}, lit.	$C_{p,m}$(l) / J mol^{-1} K^{-1}, lit.	$C_{p,m}$(l)/$C_{p,m}$(cr), lit.	$C_{p,m}$(l)/$C_{p,m}$(cr), exp.	$C_{p,m}$(l) / J mol^{-1} K^{-1}, exp.	Dev. / %
			o-terphenyl [10]			
280.0	257.2	357.6	1.390	1.394	359	0.2
285.0	262.0	360.7	1.376	1.379	361	0.2
290.0	266.9	363.8	1.363	1.366	365	0.1
295.0	271.7	367.0	1.351	1.353	368	0.2
300.0	276.5	370.2	1.339	1.340	370	0.2
305.0	281.3	373.4	1.328	1.330	374	0.2
310.0	286.1	376.7	1.317	1.320	378	0.3
			indomethacin [11]			
357.0	478.5	617.8	1.291	1.293	618	0.1
362.0	484.7	622.5	1.284	1.286	623	0.1
367.0	490.9	627.2	1.278	1.279	628	0.1
372.0	497.1	632.0	1.271	1.272	633	0.1
377.0	503.4	636.7	1.265	1.266	637	0.1
382.0	509.6	641.4	1.259	1.260	642	0.1
387.0	515.8	646.1	1.253	1.254	647	0.1
392.0	522.0	650.9	1.247	1.248	651	0.1
397.0	528.3	655.6	1.241	1.242	656	0.1
402.0	534.5	660.3	1.235	1.237	661	0.1
407.0	540.7	665.1	1.230	1.231	666	0.1

[a] Standard uncertainty $u(p)$ = 5 kPa.

Table S6
Isobaric heat capacities of supercooled liquid phases of TCB, TDAB and m-MTDAB measured in this work at 0.1 MPa [a].

T / K	$C_{p,m}$ / J mol^{-1} K^{-1}	$U(C_{p,m})$ [b] / J mol^{-1} K^{-1}	T / K	$C_{p,m}$ / J mol^{-1} K^{-1}	$U(C_{p,m})$ [b] / J mol^{-1} K^{-1}
	TCB			TDAB	
455.0	1076.0	53.8	460.0	1168.0	58.4
460.0	1085.7	54.3	465.0	1175.8	58.8
465.0	1094.8	54.7	470.0	1183.1	59.2
470.0	1103.3	55.2	475.0	1190.8	59.5
475.0	1111.2	55.6	480.0	1196.8	59.8
480.0	1121.0	56.0	485.0	1202.7	60.1
485.0	1129.4	56.5	490.0	1207.0	60.4

490.0	1138.3	56.9	492.0	1209.3	60.5
495.0	1147.3	57.4		m-MTDAB	
500.0	1155.7	57.8	367.0	1086.1	54.3
505.0	1164.6	58.2	370.0	1091.7	54.6
510.0	1173.5	58.7	375.0	1100.8	55.0
515.0	1182.7	59.1	380.0	1109.8	55.5
520.0	1191.7	59.6	385.0	1118.7	55.9
525.0	1200.6	60.0	390.0	1127.4	56.4
	TDAB		395.0	1136.0	56.8
437.0	1130.3	56.5	400.0	1144.5	57.2
440.0	1135.7	56.8	405.0	1152.9	57.6
445.0	1144.0	57.2	410.0	1161.1	58.1
450.0	1152.4	57.6	415.0	1169.2	58.5
455.0	1160.8	58.0	416.0	1170.8	58.5

[a] Standard uncertainty *u(p)* = 5 kPa;

[b] Expanded uncertainty *U* (0.95 level of confidence, k ≈ 2) of the heat capacity.

4.3 Vapor pressure measurements

The thermogravimetry – fast scanning calorimetry method is based on the evaporation/sublimation of a sample from the sensor surface at a defined temperature, with fixation of amount of sample loss Δn. Before the experiment, each sample was pre-melted to ensure reproducible thermal contact with the sensor surface. The change in sample amount was registered by including the additional segments of the absolute heat capacity measurements before and after the sublimation (Eq. S3). The vapor pressure values were calculated as follows:

$$p_{\text{sat}} = -\frac{\Delta n}{\Delta t} \cdot \frac{RT_{\text{vap}}}{S_{\text{vap}} \beta_{\text{c}}} \quad \text{(S5)},$$

where S_{vap} (m^2) is evaporation area, R is gas constant (8.314 J mol^{-1} K^{-1}), T (K) is the sensor temperature, β_{c} (m s^{-1}) is the mass-transfer coefficient, and Δt is duration of sublimation experiment.

After premelting, liquid TCB tends to crystallize to the most stable polymorph A. The polymorphic state of TCB during heat capacity and vapor pressure measurements was confirmed by subsequent melting scans. No signs of polymorphism were found for other compounds.

The evaporation area was defined based on sample images taken with an Olympus BX3M optical microscope (Japan) equipped with a 20x lens in reflection mode, before and after sublimation. The mass transfer coefficient was calculated as proposed earlier [12]. For each temperature, vapor pressure measurements were repeated at least five times.

An accuracy of the vapor pressure measurements by thermogravimetry – fast scanning calorimetry was verified using liquid phenanthrene and methyl octadecenoate. Recommended vaporization characteristics were taken from Refs. [13, 14] .

Measured values are provided in Table S5. Deviation from the recommended values differs from -6.0 to 6.7 % and lies within the estimated uncertainty of used method (Figure S3).

Table S7

Unsmoothed values of saturated vapor pressures of liquid phenanthrene and methyl octadecenoate and their comparison with recommended literature values [13, 14].

T / K	p_{exp} / Pa	$u(p)^{\text{a}}$ / Pa	p_{lit} / Pa	$100(p_{\text{exp}} - p_{\text{lit}})/p_{\text{lit}}$
		phenanthrene (liquid)		

376.1	34	7	36	-4.1
381.2	46	9	49	-6.0
386.2	62	12	65	-4.6
391.3	84	17	86	-2.9
396.4	111	22	114	-2.6
401.5	144	29	148	-3.2
406.6	187	37	192	-2.7
411.7	243	49	247	-1.6
416.8	311	62	315	-1.3
		methyl octadecenoate (liquid)		
355.7	0.58	0.12	0.57	1.4
360.8	0.92	0.18	0.90	2.9
365.9	1.5	0.3	1.4	4.1
371.0	2.1	0.4	2.1	0.4
376.1	3.4	0.7	3.2	6.7
381.2	4.6	0.9	4.8	-3.3
386.2	7.3	1.5	7.1	3.2
391.3	11	2	10	5.2
396.4	14	3	15	-1.9

[a] The estimated uncertainties (standard deviation (u)) of vapor pressure include the uncertainty of mass transfer coefficient estimation (10 %) and reproducibility of the measurements (5-10 %). The latter is contributed by the uncertainties of mass loss rate (3-5 %), uncertainty of evaporation area determination (5 %), and uncertainty of temperature (1 K). The uncertainty of mass loss rate determination includes reproducibility of the heat flow (1 %) and uncertainty of the heat capacity (1 % [15, 16]).

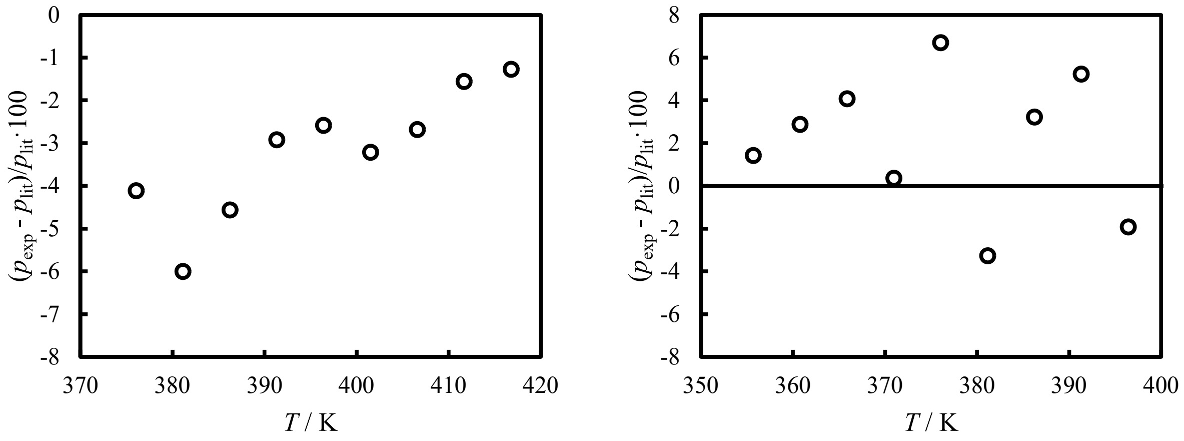

Figure S3. Deviation plots of measured vapor pressures from recommended literature values for phenanthrene (left) [13] and methyl octadecenoate (right) [14].

Vaporization enthalpies derived from p-T data were compared with literature values. For liquid phenanthrene, obtained $\Delta_l^g H(396\,\mathrm{K}) = 70.6 \pm 2.9\,\mathrm{kJ\,mol^{-1}}$ was in good agreement with literature value of 69.3 kJ mol^{-1} [13]. The reasonable agreement (93.2±2.5 kJ mol^{-1} in this work vs 91.0 kJ mol^{-1} [14] at 376 K) was also found for liquid methyl octadecenoate.

Table S8

Values of saturated vapor pressures of mCP, TCB, TDAB, and m-MTDAB measured in this work using thermogravimetry – fast scanning calorimetry.

**T / K**	**p / Pa**	**$u(p)$ [a] / Pa**	**T / K**	**p / Pa**	**$u(p)$ [a] / Pa**
	mCP, liquid			TDAB, liquid	
436.5 [b]	$9.4 \cdot 10^{-3}$	$1.8 \cdot 10^{-3}$	446.6 [b]	$7.4 \cdot 10^{-3}$	$1.5 \cdot 10^{-3}$

441.6 [b]	$1.46\cdot10^{-2}$	$0.29\cdot10^{-2}$	451.7 [b]	$1.13\cdot10^{-2}$	$0.23\cdot10^{-2}$
446.6 [b]	$2.2\cdot10^{-2}$	$0.4\cdot10^{-2}$	456.7 [b]	$1.74\cdot10^{-2}$	$0.35\cdot10^{-2}$
451.7	$3.2\cdot10^{-2}$	$0.6\cdot10^{-2}$	461.8 [b]	$2.6\cdot10^{-2}$	$0.5\cdot10^{-2}$
456.7	$4.7\cdot10^{-2}$	$0.9\cdot10^{-2}$	466.9 [b]	$4.0\cdot10^{-2}$	$0.8\cdot10^{-2}$
461.8	$6.8\cdot10^{-2}$	$1.4\cdot10^{-2}$	471.9 [b]	$6.0\cdot10^{-2}$	$1.2\cdot10^{-2}$
466.9	$9.5\cdot10^{-2}$	$1.9\cdot10^{-2}$	477.0 [b]	$9.0\cdot10^{-2}$	$1.8\cdot10^{-2}$
471.9	0.141	0.028	482.0 [b]	0.131	0.026
477.0	0.20	0.04	487.1 [b]	0.190	0.038
482.0	0.28	0.06	492.2 [b]	0.28	0.06
487.1	0.41	0.08	497.2 [b]	0.39	0.08
492.2	0.57	0.11	502.3 [b]	0.57	0.11
497.2	0.78	0.16	507.3 [b]	0.81	0.16
502.3	1.07	0.21	512.4 [b]	1.12	0.22
507.3	1.49	0.30	517.5 [b]	1.57	0.31
512.4	1.98	0.40	522.5 [b]	2.1	0.4
517.5	2.7	0.53	527.6 [b]	2.8	0.6
522.5	3.5	0.70	532.6	3.8	0.8
527.6	4.7	0.93	537.7	5.1	1.0
	mCP, crystal			TDAB, crystal	
415.7	$7.7\cdot10^{-4}$	$1.5\cdot10^{-4}$	460.8	$4.4\cdot10^{-3}$	$0.8\cdot10^{-3}$
420.7	$1.34\cdot10^{-3}$	$0.27\cdot10^{-3}$	465.8	$7.7\cdot10^{-3}$	$1.5\cdot10^{-3}$
425.7	$2.2\cdot10^{-3}$	$0.4\cdot10^{-3}$	470.9	$1.31\cdot10^{-2}$	$0.26\cdot10^{-2}$
430.7	$3.6\cdot10^{-3}$	0.00072	475.9	$2.2\cdot10^{-2}$	$0.4\cdot10^{-2}$
435.8	$6.1\cdot10^{-3}$	$1.2\cdot10^{-3}$	480.9	$3.8\cdot10^{-2}$	$0.8\cdot10^{-2}$
440.8	$1.10\cdot10^{-2}$	$0.22\cdot10^{-2}$	485.9	$5.9\cdot10^{-2}$	$1.2\cdot10^{-2}$
445.8	$1.71\cdot10^{-2}$	$0.34\cdot10^{-2}$	490.9	$9.8\cdot10^{-2}$	$2.0\cdot10^{-2}$
	TCB, liquid		495.9	0.152	0.030
485.9 [b]	$8.9\cdot10^{-4}$	$1.8\cdot10^{-4}$	500.9	0.32	0.06
495.9 [b]	$2.1\cdot10^{-3}$	$0.4\cdot10^{-3}$		m-MTDAB, liquid	
505.9 [b]	$4.9\cdot10^{-3}$	$1.0\cdot10^{-3}$	446.6 [b]	$3.6\cdot10^{-3}$	$0.7\cdot10^{-3}$
511.0 [b]	$7.0\cdot10^{-3}$	$1.4\cdot10^{-3}$	451.7 [b]	$5.6\cdot10^{-3}$	$1.1\cdot10^{-3}$
516.0 [b]	$1.07\cdot10^{-2}$	$0.21\cdot10^{-2}$	456.7 [b]	$8.9\cdot10^{-3}$	$1.8\cdot10^{-3}$
521.0 [b]	$1.54\cdot10^{-2}$	$0.31\cdot10^{-2}$	461.8	$1.36\cdot10^{-2}$	$0.27\cdot10^{-2}$
526.0 [b]	$2.3\cdot10^{-2}$	$0.5\cdot10^{-2}$	466.9	$2.1\cdot10^{-2}$	$0.4\cdot10^{-2}$
531.0 [b]	$3.6\cdot10^{-2}$	0.7	471.9	$3.3\cdot10^{-2}$	$0.7\cdot10^{-2}$
536.0 [b]	$5.4\cdot10^{-2}$	$1.1\cdot10^{-2}$	477.0	$5.0\cdot10^{-2}$	$1.0\cdot10^{-2}$
541.0 [b]	$7.2\cdot10^{-2}$	$1.4\cdot10^{-2}$	482.0	$7.4\cdot10^{-2}$	$1.5\cdot10^{-2}$
546.1 [b]	0.102	0.020	487.1	0.111	0.022
551.1 [b]	0.148	0.030	492.2	0.166	0.033
556.1 [b]	0.198	0.040	497.2	0.24	0.05
561.1 [b]	0.27	0.05	502.3	0.35	0.07
566.1 [b]	0.37	0.07	507.3	0.51	0.10
571.1 [b]	0.48	0.10	512.4	0.71	0.14
			517.5	1.00	0.20

[a] The estimated uncertainties (standard deviation (u)) of vapor pressure include the uncertainty of mass transfer coefficient estimation (10 %) and reproducibility of the measurements (5-10 %). The latter is contributed by the uncertainties of mass loss rate (3-5 %), uncertainty of evaporation area determination (5 %), and uncertainty of temperature (1 K). The uncertainty of mass loss rate determination includes reproducibility of the heat flow (1 %) and uncertainty of the heat capacity (3 %);

[b] Supercooled liquid phase.

5 Calculated vapor pressures

Vapor pressures above crystalline phases were calculated as follows. Firstly, vapor pressure above liquid mCP, TCB, TDAB, and *m*-MTDAB were extrapolated to their melting points T_m. At this temperature, $p(\mathrm{cr}) = p(\mathrm{liq})$. Further, sublimation enthalpies of each compound were found as a sum of vaporization and fusion enthalpies at their T_m. Finally, vapor pressures were extrapolated to low-temperature area according to Eq. 2. Parameters of Eq. 2 for TCB and *m*-MTDAB compounds are given in Table S10.

Table S9

Vapor pressures above crystalline phases of TCB and m-MTDAB calculated on the base of the vaporization and melting data.

T / K	p / Pa	T / K	p / Pa
TCB, crystal A		m-MTDAB	
550	$5.7{\cdot}10^{-2}$	415	$3.4{\cdot}10^{-5}$
555	$8.7{\cdot}10^{-2}$	420	$7.0{\cdot}10^{-5}$
560	0.130	425	$1.42{\cdot}10^{-4}$
565	0.194	430	$2.8{\cdot}10^{-4}$
570	0.29	435	$5.6{\cdot}10^{-4}$
575	0.42	440	$1.07{\cdot}10^{-3}$
580	0.61	445	$2.0{\cdot}10^{-3}$
585	0.89	450	$3.8{\cdot}10^{-3}$
590	1.28	455	$7.0{\cdot}10^{-3}$

Table S10

Parameters of Clarke-Glew equation (Eq. 3) used for vapor pressure calculations above crystalline phases of TCB and m-MTDAB.

Compound	T_0	$\ln(p(T_0)$ / Pa)	$\Delta_l^g H(T_0)$ / kJ mol^{-1}	$\Delta_l^g C_{p,m}$ / J mol^{-1} K^{-1}
TCB, crystal A	599.2	0.935	211.8	-62
m-MTDAB	457.1	-4.703	207.8	-76

6 Solution calorimetry

The measuring procedure was as follows: the crystalline samples with masses of 40-90 mg were placed in glass ampoules. Subsequently, the ampoules were immersed in a calorimetric cell filled with 100 mL of benzene. Thereafter, the solution enthalpy was determined after the ampoule was broken in the pre-thermostated cells. Before and after the breaking of the ampoule, electrical calibrations were performed. Infinite dilution conditions were confirmed by monitoring the concentration dependence of the solution enthalpy (molalities ranged from 0.7 to 2.5 mmol kg^{-1}). The accuracy of the technique was verified by the measurement of the solution enthalpies of propanol-1 and potassium chloride in bidistilled water (Table S11).

Table S11

Experimental enthalpies of solution of propanol-1 and potassium chloride in water measured at 298.15 K and 0.1 MPa[a].

Solute	Mass of sample [b] / mg	Molality [c] / mmol kg^{-1}	$\Delta_{soln}H^{A/S}$ [d] / kJ mol^{-1}
propanol-1	80.5	14.9	–10.20
	85.2	15.8	–10.13
	86.0	30.8	–10.10
	87.2	31.9	–10.17
		**Average**	**–10.15 ± 0.04**
		**Literature [17]**	**–10.16 ± 0.02**
KCl	36.1	4.84	17.43

65.3	8.76	17.41
66.2	8.88	17.39
62.1	8.33	17.37
	**Average**	**17.40 ± 0.03**
	**Literature [18]**	**17.47 ± 0.07**

[a] Standard uncertainties are $u(T)$ = 0.01 K, $u(p)$ = 5 kPa.
[b] Mass of solute sample which was added in each dissolution experiment.
[c] Molality of solute in solution after experiments. Standard uncertainties u are $u(b)$ = 0.01 mmol·kg^{-1}.
[d] Enthalpy of solution of each experiment.
Uncertainties of the average solution enthalpies correspond to expanded uncertainties of the mean U (0.95 level of confidence. Coverage factor $k \approx 2$).

Table S12
Experimental solution enthalpies of mCP, TCB, TDAB and m-MTDAB in benzene measured in this work at 298.15 K and 0.1MPa [a].

Compound	Mass of sample [b] / mg	Molality [c] / mmol kg^{-1}	$\Delta_{soln}H^{A/S}$ [d] / kJ mol^{-1}
mCP	40.90	1.15	14.29
	39.64	1.11	14.55
	49.83	2.54	14.09
	49.39	2.49	14.39
		**Average**	**14.3±0.2[e]**
TCB	40.63	0.81	15.65
	40.95	0.82	15.86
	49.32	1.79	15.66
	49.74	1.81	15.81
		**Average**	**15.7±0.2[e]**
TDAB	89.85	1.77	20.63
	56.96	1.12	20.55
	60.46	2.32	21.85
	56.55	1.12	21.10
	69.52	2.49	21.22
		**Average**	**21.1±0.5[e]**
m-MTDAB	38.61	0.71	27.16
	38.95	0.72	26.60
	39.83	1.44	27.13
	39.54	1.44	26.94
		**Average**	**27.0±0.4[e]**

[a] Standard uncertainties u are $u(T)$ = 0.01 K, $u(p)$ = 5 kPa;
[b] Mass of solute sample which was added in each dissolution experiment;
[c] Molality of solute in solution after experiments. Standard uncertainties u are $u(b)$ = 0.01 mmol·kg^{-1};
[d] Enthalpy of solution of each experiment;
[e] Expanded uncertainty U (0.95 level of confidence, k ≈ 2), including the reproducibility of the measurement and calibration.

7 Calculation of the internal rotation contributions to the ideal gas heat capacities

1-D hindered rotor approximation (1-DHR) was used for the determination of the internal rotation contribution to the ideal gas heat capacities of mCP, TCB, and TDAB following the algorithm described in Ref. [19]. The potential energy surface (PES) was obtained for each rotating top of the studied molecules by performing an optimized 360-degree scan with a step size of 10°. The reduced moments of inertia of the rotating tops were calculated according to the procedure proposed by Kilpatrick and Pitzer [20]. Obtained potential energy surfaces and reduced moments of inertia were used for the determination of energy levels of each internal rotation with the

Fourier grid Hamiltonian (FGH) method [21-23] using software provided by NIST [24]. The determination of internal rotation contributions to the ideal gas heat capacities of mCP, TCB, and TDAB was performed using calculated energy levels (Table S16) and Eq. S7 [19].

Table S13

Cartesian coordinates of mCP, TCB and TDAB optimized with B3LYP/6-31+G(d,p).

Atom	X, Å	Y, Å	Z, Å
		mCP	
N	–2.430495	0.219448	0.420036
N	2.430495	–0.219448	0.420036
C	–3.527996	–0.642532	0.535986
C	–2.745247	1.213613	–0.515369
C	3.527996	0.642532	0.535986
C	2.745247	–1.213613	–0.515369
C	–1.207735	0.106073	1.134779
C	1.207735	–0.106073	1.134779
C	–4.556336	–0.198171	–0.335067
C	–4.057661	0.986156	–1.004330
C	4.556336	0.198170	–0.335068
C	4.057661	–0.986156	–1.004330
C	0.000000	0.000000	0.434836
C	–1.207489	0.114345	2.535908
C	1.207489	–0.114343	2.535908
C	–3.675194	–1.793074	1.317859
C	–1.984214	2.308255	–0.938465
C	3.675195	1.793075	1.317858
C	1.984213	–2.308255	–0.938464
C	–5.757789	–0.915247	–0.406481
C	–4.604898	1.865434	–1.947922
C	5.757789	0.915247	–0.406482
C	4.604897	–1.865435	–1.947921
C	0.000000	0.000001	3.226644
C	–4.881128	–2.488780	1.228010
C	–2.552577	3.168825	–1.877835
C	4.881128	2.488780	1.228009
C	2.552577	–3.168825	–1.877834
C	–5.915954	–2.055756	0.378639
C	–3.847593	2.951125	–2.383281
C	5.915955	2.055756	0.378639
C	3.847593	–2.951126	–2.383280
H	0.000000	0.000000	–0.649633
H	–2.145276	0.218754	3.071159
H	2.145276	–0.218752	3.071159
H	–2.880825	–2.139671	1.970062
H	–0.987818	2.490002	–0.550511
H	2.880826	2.139671	1.970061
H	0.987818	–2.490002	–0.550510
H	–6.554739	–0.588327	–1.068394
H	–5.608041	1.704529	–2.332526
H	6.554739	0.588326	–1.068394
H	5.608041	–1.704531	–2.332526
H	0.000000	0.000001	4.312442
H	–5.019980	–3.384631	1.826135

Atom	X, Å	Y, Å	Z, Å
H	−1.980605	4.024819	−2.223642
H	5.019981	3.384631	1.826134
H	1.980605	−4.024819	−2.223640
H	−6.842802	−2.619275	0.332606
H	−4.259600	3.638664	−3.115577
H	6.842803	2.619274	0.332605
H	4.259600	−3.638666	−3.115575
		TCB	
N	−0.000854	2.810899	−0.000019
N	2.388079	−1.402505	0.458928
N	−2.387184	−1.403960	−0.458960
C	−0.000405	1.396560	−0.000043
C	1.188709	−0.694338	0.216874
C	−1.188239	−0.695064	−0.216935
C	0.825511	3.628175	−0.781524
C	−0.827760	3.627607	0.781505
C	3.595206	−1.223772	−0.228238
C	2.566458	−2.400483	1.426900
C	−3.594414	−1.225913	0.228200
C	−2.564970	−2.402086	−1.426887
C	1.191278	0.703804	0.233366
C	−1.191663	0.703074	−0.233447
C	0.000448	−1.398392	−0.000021
C	0.525097	4.985966	−0.497904
C	−0.528220	4.985604	0.497950
C	4.559876	−2.118461	0.303831
C	3.905074	−2.866657	1.358622
C	−4.558548	−2.121212	−0.303815
C	−3.903305	−2.869059	−1.358581
C	1.779265	3.271563	−1.737058
C	−1.781298	3.270333	1.737007
C	3.892569	−0.374099	−1.295864
C	1.664117	−2.892588	2.372785
C	−3.892281	−0.376352	1.295774
C	−1.662339	−2.893689	−2.372757
C	1.215016	5.999655	−1.172293
C	−1.218800	5.998816	1.172381
C	5.850704	−2.140791	−0.235923
C	4.335098	−3.861061	2.244403
C	−5.849357	−2.144292	0.235951
C	−4.332740	−3.863755	−2.244321
C	2.453528	4.300200	−2.394926
C	−2.456235	4.298504	2.394913
C	5.186241	−0.415157	−1.816108
C	2.117915	−3.882700	3.244486
C	−5.185925	−0.418161	1.816029
C	−2.115552	−3.884104	−3.244420
C	2.180850	5.651178	−2.114673
C	−2.184422	5.649671	2.114731
C	6.159200	−1.284623	−1.291591
C	3.436511	−4.368017	3.181153
C	−6.158361	−1.288249	1.291572

Atom	X, Å	Y, Å	Z, Å
C	−3.433857	−4.370206	−3.181060
H	2.103109	1.245636	0.452971
H	−2.103832	1.244346	−0.453032
H	0.000777	−2.481385	−0.000007
H	1.985033	2.233527	−1.968500
H	−1.986405	2.232152	1.968392
H	3.146213	0.291224	−1.712741
H	0.648755	−2.518470	2.435210
H	−3.146326	0.289455	1.712599
H	−0.647202	−2.518966	−2.435203
H	0.996467	7.043131	−0.966346
H	−1.000923	7.042442	0.966484
H	6.599626	−2.819745	0.160534
H	5.356325	−4.227792	2.203929
H	−6.597873	−2.823718	−0.160465
H	−5.353747	−4.231095	−2.203828
H	3.201637	4.049269	−3.140460
H	−3.204196	4.047054	3.140421
H	5.443176	0.237520	−2.644644
H	1.436000	−4.283855	3.987797
H	−5.443251	0.234413	2.644525
H	−1.433402	−4.284878	−3.987720
H	2.724711	6.427576	−2.642983
H	−2.728794	6.425693	2.643068
H	7.157181	−1.290586	−1.717842
H	3.756052	−5.140679	3.872978
H	−7.156337	−1.294786	1.717827
H	−3.752941	−5.143085	−3.872854
	TDAB		
N	2.382959	−1.407457	0.539281
N	−2.382497	−1.408035	−0.539419
N	−0.000343	2.801387	0.000062
C	1.178136	−0.711890	0.274504
C	−1.177832	−0.712183	−0.274599
C	−0.000128	1.387166	−0.000020
C	0.000233	−1.418084	−0.000051
C	1.170935	0.687494	0.314331
C	−1.171006	0.687202	−0.314396
C	3.609823	−0.901373	0.041196
C	2.362095	−2.615379	1.281369
C	−3.609425	−0.902376	−0.041056
C	−2.361337	−2.615899	−1.281563
C	−1.150113	3.511915	0.431030
C	1.149038	3.512456	−0.430985
C	4.768186	−0.942426	0.831822
C	1.560478	−2.729057	2.427179
C	−4.767895	−0.943656	−0.831527
C	−3.139913	−3.710565	−0.878154
C	−1.870045	3.085049	1.557189
C	1.869478	3.085658	−1.556852
C	3.680271	−0.348224	−1.246892
C	3.140803	−3.709875	0.877752

Atom	X, Å	Y, Å	Z, Å
C	−3.679837	−0.349387	1.247095
C	−1.559522	−2.729361	−2.427267
C	−1.583367	4.647530	−0.269081
C	1.581396	4.648656	0.268751
C	5.972549	−0.442565	0.338069
C	1.532633	−3.922034	3.147697
C	−5.972319	−0.444184	−0.337551
C	−3.120010	−4.894405	−1.614772
C	−3.011324	3.774425	1.962810
C	3.010354	3.775670	−1.962529
C	4.883498	0.167073	−1.725014
C	3.121215	−4.893775	1.614277
C	−4.883144	0.165533	1.725444
C	−1.531365	−3.922273	−3.147869
C	−2.714327	5.343786	0.155623
C	2.711946	5.345528	−0.156024
C	6.038479	0.120748	−0.939434
C	2.314653	−5.010375	2.749763
C	−6.038219	0.118974	0.940030
C	−2.313263	−5.010784	−2.750144
C	−3.439261	4.909751	1.269082
C	3.437388	4.911565	−1.269180
H	0.000367	−2.500255	−0.000075
H	2.074666	1.224696	0.569690
H	−2.074891	1.224167	−0.569708
H	4.716654	−1.365983	1.828990
H	0.959558	−1.882321	2.740574
H	−4.716373	−1.367084	−1.828750
H	−3.757453	−3.625130	0.009425
H	−1.535026	2.208636	2.100446
H	1.535173	2.208824	−2.099861
H	2.787554	−0.319549	−1.861612
H	3.758194	−3.624246	−0.009913
H	−2.787042	−0.320508	1.861691
H	−0.958701	−1.882498	−2.740507
H	−1.029657	4.977631	−1.141322
H	1.027316	4.978722	1.140767
H	6.859942	−0.479731	0.962974
H	0.905361	−3.996402	4.031117
H	−6.859796	−0.481513	−0.962328
H	−3.727432	−5.733917	−1.289808
H	−3.562856	3.426486	2.830989
H	3.562276	3.427769	−2.830475
H	4.919914	0.596471	−2.721860
H	3.728725	−5.733165	1.289166
H	−4.919524	0.594819	2.722340
H	−0.903949	−3.996466	−4.031202
H	−3.038502	6.220621	−0.397001
H	3.035407	6.222804	0.396319
H	6.975070	0.518996	−1.316283
H	2.295065	−5.936006	3.316094
H	−6.974871	0.516920	1.317046

Atom	X, Å	Y, Å	Z, Å
H	–2.293429	–5.936361	–3.316553
H	–4.325960	5.446872	1.590180
H	4.323769	5.449172	–1.590340

Table S14

Reduced moments of inertia I_r of the rotating tops of mCP, TCB and TDAB.

Rotating top	I_r / amu Å^2
mCP	
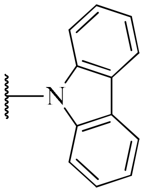	566.488 [a]
TCB	
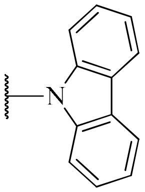	757.922±0.161 [b]
TDAB	
$–NPh_2$	976.166±1.655 [b]
–Ph	87.701±0.058 [b]

[a] The difference between the reduced moments of inertia of the identical groups is negligible.

[b] Average reduced moment of inertia of all the groups of a particular type in a molecule and standard deviation of the mean.

Table S15

Computed fundamental vibrational wavenumbers of mCP, TCB and TDAB used in the calculation of the ideal-gas heat capacities. The frequencies corresponding to the internal rotation were identified according to Ayala [25] and excluded from the table and further calculations.

ν / cm^{-1}	ν / cm^{-1}	ν / cm^{-1}	ν / cm^{-1}	ν / cm^{-1}
		mCP		
24.5	565.2	920.4	1167.7	1586.6
52.4	567.1	922.9	1187.4	1587.2
64.3	572.7	923.2	1226.4	1597.2
74.9	572.8	925.1	1226.6	1606.2
102.1	611.3	926.1	1228.5	1609.7
110.2	615.5	927.7	1241.1	1610.8
144.5	622.0	930.0	1272.0	1631.0
146.6	634.5	960.2	1309.4	1631.8
147.5	644.9	960.3	1313.5	3043.8
197.8	668.6	961.7	1314.4	3043.8
211.1	700.2	961.7	1317.1	3044.6
239.3	700.8	975.1	1322.8	3044.6
240.2	722.7	995.0	1343.2	3051.9
276.0	722.8	996.8	1353.2	3051.9
286.0	731.7	996.9	1355.3	3053.2
286.3	735.7	999.9	1357.7	3053.2
299.4	735.8	1020.3	1359.6	3055.3
336.5	745.6	1020.6	1374.9	3065.0

ν / cm-1	ν / cm-1	ν / cm-1	ν / cm-1	ν / cm-1
369.0	745.8	1028.5	1440.0	3065.0
425.0	751.1	1028.9	1448.7	3065.4
425.2	769.1	1087.3	1457.3	3065.5
430.1	769.7	1097.0	1458.8	3072.7
430.2	780.1	1105.3	1463.3	3073.5
444.1	792.6	1119.4	1479.8	3074.0
446.0	844.5	1119.5	1482.6	3074.2
446.5	844.9	1145.9	1490.1	3075.0
457.9	849.0	1152.9	1495.3	3079.1
519.2	849.0	1157.0	1500.7	3080.3
522.8	885.6	1159.9	1585.4	
544.4	890.8	1159.9	1586.5	
		TCB		
15.2	547.9	885.2	1160.8	1589.7
20.0	547.9	889.4	1160.8	1593.7
27.9	561.3	917.4	1201.1	1593.9
44.0	570.4	918.8	1228.0	1594.1
69.5	571.3	920.2	1228.2	1605.1
72.5	571.4	920.6	1230.7	1606.7
97.6	571.6	922.1	1231.0	1613.4
98.6	589.8	923.8	1232.2	1616.2
105.9	609.8	925.2	1254.2	1616.6
134.5	619.3	927.0	1255.5	1635.9
142.1	619.7	927.9	1309.2	1636.3
144.3	623.7	929.2	1309.9	1636.7
148.1	649.9	956.5	1314.2	3047.2
151.8	650.3	957.5	1314.5	3047.4
152.9	667.9	959.0	1315.3	3047.4
210.0	672.0	959.5	1316.9	3048.1
215.5	688.9	960.9	1328.0	3048.2
241.4	706.1	961.3	1342.4	3048.3
251.7	716.1	1001.6	1342.4	3055.4
254.9	719.9	1002.1	1359.3	3055.5
277.8	723.0	1002.2	1360.1	3055.7
283.7	730.2	1002.9	1363.0	3056.6
284.1	732.3	1021.1	1363.1	3056.7
285.4	733.4	1022.3	1363.6	3056.9
289.5	741.8	1023.1	1363.7	3069.0
331.2	741.8	1027.8	1417.5	3069.0
335.2	742.5	1028.0	1446.0	3069.2
380.0	744.0	1029.9	1446.6	3069.5
380.7	744.0	1036.0	1457.1	3069.9
419.9	755.1	1036.4	1457.7	3070.1
422.7	763.3	1103.4	1460.2	3078.2
423.8	767.7	1104.9	1465.7	3078.7
432.6	769.1	1108.7	1470.9	3081.6
433.9	790.1	1118.9	1471.6	3081.9
434.1	790.2	1120.6	1484.5	3085.3
441.5	836.2	1121.3	1488.9	3085.6
444.8	839.4	1148.2	1489.1	3089.9
445.9	843.7	1149.2	1496.6	3090.0
487.3	844.9	1154.3	1497.0	3090.8

ν / cm^{-1}	ν / cm^{-1}	ν / cm^{-1}	ν / cm^{-1}	ν / cm^{-1}
525.1	846.5	1157.0	1499.8	
525.9	848.2	1158.3	1589.0	
528.8	870.1	1160.6	1589.2	
		TDAB		
14.8	613.1	924.9	1175.2	1597.4
21.8	616.7	932.8	1176.1	1598.5
22.5	616.8	957.0	1176.4	1599.1
40.4	618.2	957.7	1177.5	1599.3
62.6	620.0	959.7	1177.8	1607.6
65.3	620.5	960.1	1214.6	1608.3
71.3	621.6	960.7	1257.9	1609.5
73.0	628.7	961.2	1258.3	1620.2
81.1	629.6	972.5	1259.6	1622.4
150.1	650.8	972.9	1286.6	1622.5
150.7	659.9	974.3	1290.3	3042.4
204.6	667.2	974.4	1291.4	3042.5
212.7	689.8	974.7	1308.5	3042.7
226.8	691.0	974.9	1309.5	3042.7
236.6	691.7	995.0	1314.7	3043.0
237.4	692.8	995.1	1322.5	3043.2
240.0	693.3	995.2	1327.3	3049.3
247.0	695.1	995.6	1327.5	3049.3
253.6	704.1	995.8	1333.2	3049.7
254.9	740.4	996.0	1333.4	3049.7
261.1	740.8	996.0	1333.8	3050.2
310.9	748.9	1025.8	1334.7	3050.3
312.1	749.8	1026.0	1336.2	3063.4
320.4	750.4	1029.9	1336.3	3063.6
362.1	750.5	1030.8	1345.4	3063.8
367.1	769.8	1031.0	1377.0	3063.9
389.1	770.9	1031.1	1447.3	3064.2
409.7	823.6	1046.2	1447.7	3064.4
410.5	825.1	1048.2	1455.4	3069.3
411.2	826.6	1083.9	1462.9	3069.6
413.6	828.2	1084.0	1463.1	3069.9
414.8	830.1	1084.6	1467.0	3070.1
415.3	830.9	1087.2	1473.8	3070.5
419.0	849.5	1087.8	1474.0	3070.7
424.6	855.5	1088.1	1498.1	3072.7
494.1	856.4	1156.3	1498.4	3072.9
499.4	876.0	1156.5	1499.1	3073.3
500.7	892.1	1156.7	1500.4	3073.4
501.0	894.0	1157.5	1500.9	3074.2
508.6	894.5	1157.7	1501.9	3074.3
510.2	896.9	1158.1	1585.7	3090.4
515.2	898.6	1158.2	1586.1	3090.6
576.6	912.3	1158.6	1590.5	3090.7
588.1	923.1	1174.9	1596.7	

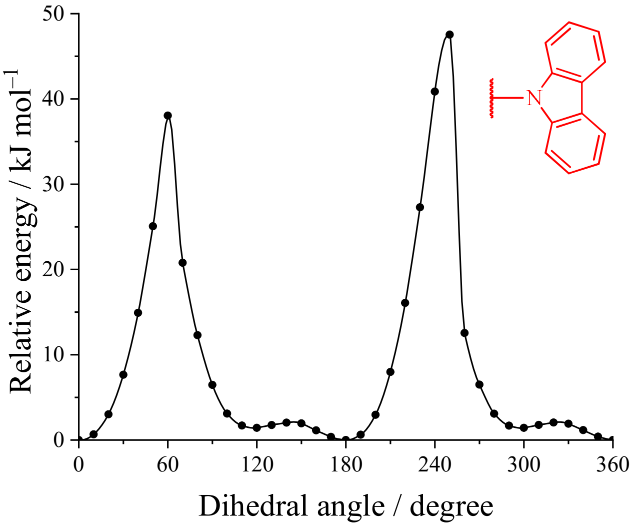

Figure S4. Potential energy surface for internal rotation in mCP. A dihedral angle of 0 degrees corresponds to the optimal configuration.

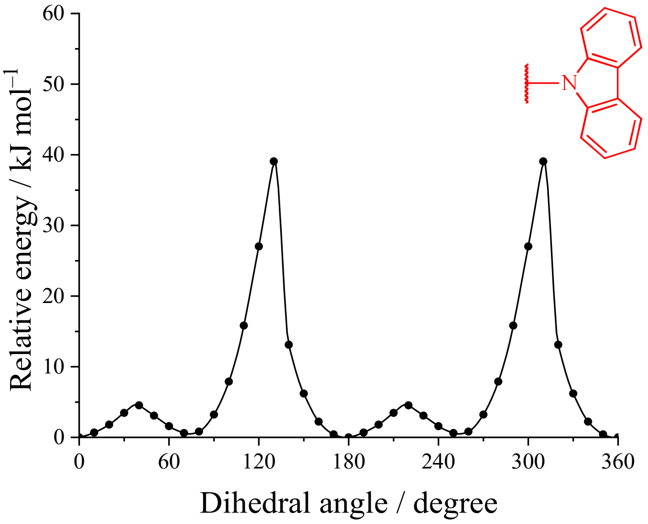

Figure S5. Potential energy surface for internal rotation in TCB. A dihedral angle of 0 degrees corresponds to the optimal configuration.

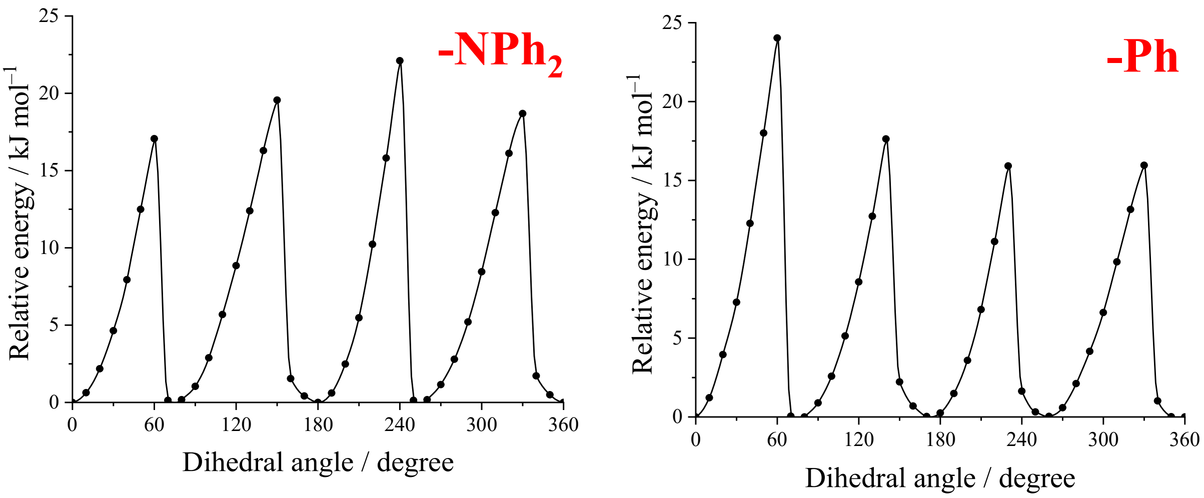

Figure S6. Potential energy surfaces for internal rotations in TDAB. A dihedral angle of 0 degrees corresponds to the optimal configuration.

Table S16

Energy levels of hindered rotors encountered in mCP, TCB and TDAB (up to a frequency of 3000 cm^{-1}).

**ν / cm^{-1}**	**ν / cm^{-1}**	**ν / cm^{-1}**	**ν / cm^{-1}**	**ν / cm^{-1}**
		mCP		
		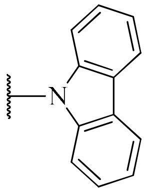		
6.3	309.1	692.4	1181.5	1746.9
8.5	310.8	697.4	1187.0	1750.7
18.7	316.6	702.0	1192.7	1760.3
22.0	318.0	707.5	1199.2	1764.3
30.9	324.1	711.6	1204.0	1774.3
33.3	325.4	717.6	1211.4	1776.0
42.7	331.8	721.3	1215.2	1789.6
44.7	332.8	727.8	1223.7	1789.6
54.4	339.5	731.1	1226.5	1800.7
56.0	340.3	738.1	1236.0	1804.7
65.9	347.4	741.0	1237.9	1814.1
67.3	347.9	748.3	1248.4	1817.0
77.2	355.3	750.9	1249.3	1828.4
78.5	355.6	758.6	1260.7	1831.8
88.5	363.3	761.0	1260.8	1842.3
89.6	363.3	769.0	1272.1	1846.8
99.7	371.2	771.1	1273.2	1852.5
100.7	371.4	779.4	1283.6	1863.7
110.8	379.1	781.2	1285.7	1869.1
111.6	379.5	789.8	1295.1	1872.1
121.8	387.0	791.5	1298.2	1890.5
122.3	387.8	800.3	1306.7	1891.6
123.6	395.1	801.8	1310.7	1894.9
125.0	396.1	810.8	1318.3	1912.5
132.7	403.2	812.1	1323.3	1913.7
132.8	404.5	821.3	1330.0	1915.5
134.2	411.4	822.6	1335.9	1937.1
135.3	413.0	831.9	1341.7	1941.2
143.0	419.7	833.1	1348.5	1946.4
143.3	421.5	842.5	1353.4	1960.1
144.2	428.1	843.6	1361.2	1970.8
144.5	430.1	853.2	1365.2	1975.7
152.8	436.5	854.3	1373.9	1982.4
153.1	438.7	863.8	1377.0	1991.1
153.6	445.0	865.0	1386.7	2008.7
153.6	447.4	874.5	1388.9	2014.5
161.1	453.5	875.7	1399.5	2025.4
162.0	456.2	885.3	1400.8	2042.9
162.0	462.1	886.6	1412.3	2059.9
163.3	465.0	896.0	1412.8	2076.5
168.5	470.8	897.4	1424.7	2088.1

ν / cm⁻¹	ν / cm⁻¹	ν / cm⁻¹	ν / cm⁻¹	ν / cm⁻¹
169.3	473.8	906.8	1425.2	2096.8
170.2	479.6	908.4	1436.8	2113.7
171.9	482.7	917.6	1438.0	2132.2
174.6	488.4	919.4	1448.9	2152.5
175.6	491.6	928.4	1451.0	2170.2
177.7	497.3	930.4	1461.0	2192.3
179.5	500.5	939.3	1464.0	2194.0
181.9	506.2	941.5	1473.1	2209.9
183.6	509.4	950.2	1477.0	2229.5
186.2	515.3	952.7	1485.3	2256.5
188.1	518.3	961.1	1490.0	2268.6
190.8	524.3	963.9	1497.6	2269.8
192.9	526.3	972.0	1503.2	2291.7
195.6	528.9	975.2	1509.9	2324.6
197.8	533.5	982.9	1516.3	2328.8
200.7	536.3	986.6	1522.2	2356.7
203.0	542.7	993.9	1529.5	2390.3
205.9	544.9	998.0	1534.5	2396.3
208.3	551.9	1004.8	1542.7	2404.3
211.3	553.2	1009.4	1546.9	2424.5
213.8	561.1	1015.8	1556.0	2452.9
216.8	561.2	1020.9	1559.4	2471.6
219.5	568.7	1026.8	1569.4	2495.0
222.6	570.6	1032.5	1571.9	2516.8
225.3	576.4	1037.8	1582.7	2550.7
228.5	580.0	1044.1	1584.4	2568.1
231.2	584.5	1048.8	1596.1	2581.8
234.5	589.5	1055.7	1597.0	2607.9
237.2	593.0	1059.8	1609.6	2633.2
240.7	599.1	1067.4	1609.6	2643.6
243.4	601.6	1070.8	1622.2	2648.0
247.0	608.7	1079.2	1623.0	2715.5
249.7	610.2	1081.8	1634.9	2718.3
253.4	618.3	1091.0	1636.6	2721.5
256.0	619.0	1092.8	1647.6	2784.2
259.9	627.8	1102.8	1650.2	2801.2
262.5	628.0	1103.8	1660.4	2806.3
266.6	636.8	1114.7	1663.7	2832.9
269.1	637.8	1114.9	1673.2	2854.2
273.4	645.8	1125.9	1677.6	2882.2
275.8	647.6	1126.6	1686.0	2898.4
280.3	654.9	1137.0	1691.1	2925.6
282.6	657.5	1138.6	1698.8	2963.8
287.4	664.2	1148.1	1705.0	2994.3
289.5	667.4	1150.7	1711.8	2998.4
294.5	673.5	1159.2	1718.9	
296.5	677.3	1162.7	1724.9	
301.8	682.9	1170.4	1732.5	
303.6	687.3	1174.8	1737.5	
		TCB		

ν / cm-1	ν / cm-1	ν / cm-1	ν / cm-1	ν / cm-1
		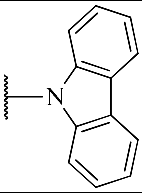		
5.8	297.3	573.7	945.3	1411.1
8.6	301.6	577.7	952.4	1420.9
18.3	303.3	580.3	954.3	1421.3
21.8	303.3	584.4	961.5	1424.0
30.1	308.1	587.1	963.3	1429.9
32.6	312.5	591.2	970.6	1447.2
41.4	315.4	593.8	972.4	1448.8
42.7	315.4	598.0	979.8	1453.9
47.0	318.9	600.7	981.6	1454.7
47.0	323.4	604.9	989.0	1466.9
52.3	327.2	607.6	990.7	1476.7
52.9	327.3	611.9	998.2	1485.9
61.2	329.6	614.6	1000.0	1489.2
61.2	334.1	618.9	1007.5	1489.5
62.7	339.0	621.6	1009.2	1506.0
63.1	339.0	626.0	1016.8	1506.1
72.8	340.1	628.7	1018.5	1526.3
73.7	344.7	633.2	1026.2	1535.6
75.2	350.5	635.8	1027.8	1536.0
75.2	350.5	640.4	1035.6	1537.0
82.8	350.5	643.0	1037.2	1547.2
84.2	355.0	647.7	1045.0	1568.3
89.0	360.6	650.3	1046.6	1568.6
89.0	361.7	655.0	1054.5	1589.4
93.1	361.7	657.6	1056.0	1589.6
94.8	365.0	662.4	1064.0	1590.0
102.5	370.2	665.0	1065.5	1601.7
102.5	372.1	669.8	1073.5	1611.7
103.3	372.4	672.4	1075.0	1634.1
105.3	374.7	677.3	1083.1	1636.5
113.6	379.0	679.9	1084.5	1656.9
115.8	381.2	684.9	1092.7	1673.6
115.9	382.6	687.5	1094.1	1680.1
115.9	384.7	692.5	1102.4	1685.2
124.0	388.0	695.1	1103.8	1686.1
126.4	390.6	700.2	1112.1	1703.8
129.2	393.0	702.7	1113.4	1713.6
129.2	395.4	707.9	1121.8	1728.0
134.5	398.4	710.4	1123.0	1752.6
137.1	401.0	715.7	1131.5	1753.7
142.2	403.9	718.2	1132.8	1777.8
142.2	406.5	723.5	1141.3	1791.3
145.1	409.5	726.0	1142.6	1803.4
147.8	412.3	731.3	1151.1	1829.5
155.1	415.2	733.8	1152.3	1830.0
155.1	418.1	739.3	1161.0	1856.3
155.7	421.1	741.7	1162.1	1877.4

ν / cm-1	ν / cm-1	ν / cm-1	ν / cm-1	ν / cm-1
158.5	424.0	747.2	1170.9	1883.6
166.4	427.0	749.7	1172.1	1911.5
167.8	430.0	755.3	1180.8	1936.6
167.8	433.0	757.7	1181.8	1939.9
169.3	436.0	763.3	1190.7	1968.9
177.2	439.1	765.7	1191.7	1998.4
180.2	442.2	771.4	1200.7	2004.9
180.4	445.2	773.8	1201.8	2028.6
180.4	448.4	779.6	1210.7	2059.5
188.0	451.4	782.0	1211.8	2080.0
191.2	454.7	787.8	1220.8	2090.9
192.9	457.7	790.2	1221.6	2123.0
192.9	461.0	796.1	1230.8	2155.8
198.9	464.1	798.4	1231.8	2160.2
202.2	467.4	804.4	1240.9	2189.3
205.4	470.4	806.7	1242.0	2223.6
205.4	473.8	812.7	1251.2	2244.3
209.8	476.9	815.0	1251.8	2258.6
213.2	480.3	821.1	1261.4	2294.4
217.8	483.3	823.4	1261.8	2331.0
217.8	486.8	829.6	1271.2	2331.2
220.7	489.8	831.8	1272.4	2368.5
224.3	493.3	838.1	1281.4	2406.8
230.1	496.3	840.3	1282.6	2420.0
230.1	499.9	846.6	1292.2	2446.0
231.7	502.9	848.8	1292.3	2486.1
235.3	506.5	855.2	1302.3	2509.8
242.4	509.4	857.4	1302.6	2527.1
242.4	513.1	863.8	1311.6	2569.0
242.6	516.0	866.0	1313.7	2599.9
246.4	519.6	872.5	1322.7	2611.9
253.6	522.5	874.6	1323.3	2655.7
254.6	526.1	881.2	1332.4	2689.6
254.6	529.0	883.3	1334.5	2700.6
257.5	532.6	889.9	1342.4	2746.5
264.6	535.4	892.0	1344.6	2778.3
266.9	539.0	898.7	1351.7	2793.3
266.9	541.7	900.8	1355.8	2841.2
268.6	545.4	907.6	1362.1	2865.1
275.5	548.0	909.6	1366.4	2889.9
279.1	551.7	916.5	1372.4	2939.3
279.1	554.3	918.4	1376.6	2949.5
279.6	558.0	925.4	1380.2	2989.3
286.4	560.7	927.3	1389.8	
290.7	564.5	934.3	1395.2	
291.2	567.1	936.3	1397.3	
291.2	571.0	943.4	1401.1	
		TDAB		
		–NPh_2		
1.3	327.7	676.1	1025.3	1502.3
1.4	330.8	678.1	1033.3	1509.7
6.5	331.7	683.5	1042.0	1522.5

ν / cm-1	ν / cm-1	ν / cm-1	ν / cm-1	ν / cm-1
11.3	333.6	689.7	1042.7	1530.0
17.9	342.9	690.9	1044.1	1536.2
20.8	344.1	691.2	1050.2	1542.0
27.4	346.7	698.2	1053.8	1550.5
27.8	346.8	703.7	1059.2	1558.4
33.6	356.5	705.0	1060.0	1562.2
34.5	358.1	706.2	1063.0	1566.1
42.7	360.1	712.8	1066.6	1578.8
45.2	362.6	716.3	1071.2	1578.9
47.7	368.9	719.6	1075.0	1586.3
50.9	373.2	721.3	1076.9	1587.4
55.7	373.6	727.4	1082.3	1589.9
58.1	378.4	728.9	1089.3	1591.5
60.8	381.5	733.6	1091.9	1607.0
62.0	387.1	736.3	1093.0	1616.8
67.8	388.2	741.7	1097.6	1622.9
70.3	394.0	742.2	1108.3	1632.1
77.5	394.2	747.9	1111.4	1634.5
78.1	400.7	751.4	1113.3	1646.6
79.2	403.3	754.3	1115.1	1649.8
80.4	406.7	757.4	1116.5	1657.8
85.3	409.9	763.3	1118.7	1662.1
89.5	414.5	766.5	1124.5	1676.3
90.0	418.3	766.8	1130.1	1679.5
94.5	419.4	772.7	1131.6	1680.5
99.5	425.5	779.6	1135.6	1684.7
100.1	428.3	779.6	1139.6	1694.8
101.4	432.1	781.6	1141.2	1703.8
104.0	433.3	787.8	1147.4	1704.7
105.7	441.1	792.4	1158.9	1705.5
111.1	442.2	796.2	1161.2	1727.4
114.0	445.0	796.7	1164.0	1728.1
114.2	448.3	801.8	1165.2	1730.7
118.2	456.2	805.0	1165.4	1736.7
122.4	456.7	811.6	1177.3	1745.2
124.2	457.8	813.1	1183.9	1752.0
128.0	463.3	814.6	1188.1	1766.0
130.9	470.4	818.0	1191.9	1777.5
133.9	470.7	826.0	1196.3	1778.7
134.7	472.2	828.6	1202.9	1781.8
142.3	478.3	829.3	1215.8	1790.2
144.7	483.6	831.3	1216.2	1806.8
145.3	484.6	840.5	1219.9	1815.9
145.6	487.6	841.0	1222.3	1825.6
156.2	493.3	844.3	1228.9	1827.2
157.2	496.6	844.4	1235.8	1834.4
157.5	498.9	851.4	1242.4	1837.1
159.1	502.9	855.3	1245.4	1843.3
167.1	508.3	857.1	1249.1	1869.0
169.4	509.6	860.7	1249.8	1873.2
172.3	513.3	867.5	1256.2	1875.9
173.9	518.2	870.0	1262.9	1891.9

ν / cm⁻¹	ν / cm⁻¹	ν / cm⁻¹	ν / cm⁻¹	ν / cm⁻¹
178.3	522.6	870.5	1267.4	1903.6
181.6	523.3	877.3	1274.2	1904.7
187.8	527.8	882.7	1275.7	1915.4
189.0	533.4	884.7	1277.2	1925.8
189.6	535.6	886.0	1279.9	1939.1
193.8	538.3	893.3	1283.9	1939.9
200.9	542.4	895.5	1298.8	1961.2
203.4	548.6	901.2	1305.6	1961.8
204.3	548.6	902.2	1307.2	1975.4
206.1	553.4	903.3	1312.1	1979.6
212.4	557.0	909.5	1320.9	1995.5
218.5	561.7	914.0	1327.8	2015.2
219.2	563.8	914.6	1333.8	2021.5
219.8	568.5	919.9	1339.7	2034.2
224.0	571.7	924.5	1343.7	2038.0
231.0	574.7	925.9	1345.9	2057.1
235.0	578.8	930.3	1350.7	2068.2
235.3	583.5	937.5	1367.1	2080.3
235.7	586.5	939.1	1368.7	2104.8
243.5	587.7	939.7	1373.1	2118.4
247.4	593.9	947.3	1374.2	2130.7
250.8	598.5	952.0	1381.6	2134.8
251.0	600.7	955.0	1383.2	2156.6
256.1	601.3	955.0	1391.3	2178.0
259.2	608.8	963.1	1398.4	2190.0
266.3	613.5	964.4	1407.2	2219.7
267.0	613.8	970.2	1416.2	2245.1
268.8	616.2	970.3	1418.8	2246.1
271.2	623.8	974.0	1419.1	2255.0
281.6	626.7	974.1	1423.3	2291.2
281.8	628.5	981.5	1427.2	2332.2
282.9	631.1	986.6	1441.3	2332.5
283.1	638.7	988.3	1441.8	2383.3
294.5	639.6	990.4	1449.0	2418.1
295.2	643.5	998.5	1458.3	2425.0
297.1	646.1	1004.5	1468.1	2434.2
298.9	652.6	1005.2	1474.1	2490.3
307.3	653.7	1007.6	1475.3	2525.2
307.4	658.8	1007.8	1483.6	2600.3
312.5	661.1	1016.4	1486.6	2648.2
314.9	665.4	1017.6	1495.0	2663.5
319.5	668.6	1023.3	1495.0	2845.2
320.4	674.2	1024.9	1499.3	
		–Ph		
5.8	462.8	967.3	1494.0	2139.3
7.2	468.6	978.3	1499.7	2166.6
13.3	472.7	981.6	1512.4	2170.9
22.8	490.1	996.9	1528.7	2200.4
29.2	504.8	1017.1	1542.3	2203.2
40.1	511.8	1019.7	1552.5	2234.6
57.1	516.4	1025.5	1563.4	2236.4
57.8	542.8	1049.6	1578.3	2269.2

ν / cm^{-1}	ν / cm^{-1}	ν / cm^{-1}	ν / cm^{-1}	ν / cm^{-1}
62.2	546.7	1058.5	1592.5	2270.4
92.8	551.1	1066.0	1605.1	2304.3
92.8	564.7	1073.3	1617.3	2305.0
93.1	588.5	1097.9	1631.1	2339.8
94.8	590.8	1103.5	1645.5	2340.3
123.1	595.6	1113.9	1659.3	2375.8
126.7	613.5	1121.5	1672.7	2376.1
127.3	629.9	1137.9	1686.6	2412.2
136.6	630.9	1158.5	1701.0	2412.4
154.4	648.0	1160.8	1715.4	2449.0
163.5	663.2	1170.1	1729.6	2449.1
178.5	671.1	1178.3	1743.8	2486.3
181.9	671.2	1207.4	1758.5	2486.4
188.7	699.5	1214.5	1773.2	2524.1
201.3	711.6	1218.4	1788.0	2524.1
224.7	712.6	1219.3	1802.6	2562.2
228.4	713.3	1254.0	1817.7	2562.3
230.8	749.7	1259.7	1832.5	2600.8
239.7	751.9	1267.2	1847.8	2600.8
261.9	754.7	1271.3	1862.7	2639.9
276.5	763.9	1299.5	1878.2	2639.9
278.5	791.8	1301.3	1893.1	2679.3
283.1	797.7	1314.8	1909.0	2679.3
300.2	798.4	1328.5	1923.8	2719.2
317.4	815.0	1339.3	1940.1	2719.2
324.8	831.0	1347.6	1954.7	2759.5
334.6	841.5	1360.9	1971.5	2759.5
339.6	846.6	1378.7	1985.6	2800.2
356.4	865.9	1385.7	2003.2	2800.2
373.0	869.4	1393.3	2016.5	2841.3
380.0	886.3	1404.8	2035.2	2841.3
386.1	895.4	1420.3	2047.3	2882.9
395.2	906.9	1437.4	2067.5	2882.9
421.1	916.8	1442.3	2077.9	2924.8
421.1	931.9	1449.6	2100.2	2924.8
434.0	944.1	1464.9	2108.5	2967.2
437.8	945.5	1482.0	2133.2	2967.2

Table S17

Contributions of internal rotation to the heat capacity of mCP, TCB and TDAB.

T / K	$C_{v,ir}(T)$ / J K^{-1} mol^{-1}	$C_{v,ir}(T)$ / J K^{-1} mol^{-1}
	mCP	TCB
Rotating top	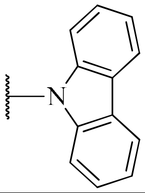	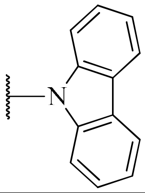
200	7.0	7.7
220	6.8	7.5
240	6.6	7.3
260	6.5	7.1
280	6.4	6.9

T / K	$C_{v,ir}(T)$ / J K^{-1} mol^{-1}	$C_{v,ir}(T)$ / J K^{-1} mol^{-1}
298.15	6.3	6.8
300	6.3	6.8
320	6.2	6.6
340	6.1	6.4
360	6.1	6.3
380	6.0	6.1
400	5.9	6.0
420	5.9	5.8
440	5.8	5.7
460	5.7	5.5
480	5.7	5.4
500	5.6	5.3
520	5.5	5.1
540	5.5	5.0
560	5.4	4.9
580	5.3	4.8
600	5.3	4.7
	TDAB	
Rotating top	–NPh_2	–Ph
200	7.7	7.5
220	7.7	7.5
240	7.7	7.5
260	7.6	7.6
280	7.5	7.6
298.15	7.5	7.6
300	7.5	7.6
320	7.4	7.6
340	7.3	7.6
360	7.2	7.6
380	7.0	7.5
400	6.9	7.5
420	6.8	7.5
440	6.6	7.5
460	6.5	7.4
480	6.4	7.4
500	6.2	7.3
520	6.1	7.3
540	6.0	7.2
560	5.8	7.2
580	5.7	7.1
600	5.5	7.1

Table S18

Contributions of vibration and internal rotation to the heat capacities of mCP, TCB and TDAB, as well as isochoric and isobaric heat capacities calculated in this work.

T / K	$C_{v,vib}(T)$ / J K^{-1} mol^{-1} [a]	$\sum C_{v,ir}(T)$ / J K^{-1} mol^{-1} [b]	$C_{v,m}(T)$ / J K^{-1} mol^{-1}	$C_{p,m}(T)$ / J K^{-1} mol^{-1}
		mCP		
200	223.2	14.1	262.2	270.5
220	252.2	13.6	290.8	299.1
240	282.0	13.2	320.2	328.5
260	312.2	13.0	350.1	358.4
280	342.4	12.7	380.1	388.4

T / K	$C_{v,vib}(T)$ / J K^{-1} mol^{-1} [a]	$\sum C_{v,ir}(T)$ / J K^{-1} mol^{-1} [b]	$C_{v,m}(T)$ / J K^{-1} mol^{-1}	$C_{p,m}(T)$ / J K^{-1} mol^{-1}
298.15	369.7	12.6	407.2	415.5
300	372.5	12.6	410.0	418.3
320	402.1	12.4	439.5	447.8
340	431.2	12.2	468.4	476.7
360	459.5	12.1	496.5	504.8
380	486.9	12.0	523.8	532.1
400	513.4	11.8	550.2	558.5
420	538.9	11.7	575.5	583.8
440	563.3	11.6	599.9	608.2
460	586.7	11.5	623.2	631.5
480	609.1	11.3	645.4	653.7
500	630.6	11.2	666.7	675.0
520	651.0	11.1	687.0	695.3
540	670.5	11.0	706.4	714.7
560	689.1	10.8	724.9	733.2
580	706.9	10.7	742.5	750.8
600	723.9	10.5	759.3	767.7
		TCB		
200	325.3	23.2	373.5	381.8
220	366.0	22.6	413.5	421.8
240	407.5	22.0	454.4	462.7
260	449.5	21.4	495.8	504.1
280	491.6	20.8	537.4	545.7
298.15	529.6	20.3	574.9	583.2
300	533.5	20.3	578.7	587.0
320	574.7	19.8	619.4	627.7
340	615.1	19.3	659.3	667.6
360	654.4	18.8	698.1	706.4
380	692.5	18.3	735.7	744.0
400	729.3	17.9	772.0	780.4
420	764.6	17.4	807.0	815.3
440	798.6	17.0	840.5	848.8
460	831.1	16.6	872.6	880.9
480	862.2	16.2	903.3	911.7
500	892.0	15.8	932.7	941.0
520	920.4	15.4	960.7	969.0
540	947.5	15.0	987.4	995.7
560	973.3	14.6	1012.9	1021.2
580	998.0	14.3	1037.2	1045.5
600	1021.5	14.0	1060.4	1068.8
		TDAB		
200	311.9	68.2	405.0	413.3
220	353.4	68.2	446.6	454.9
240	396.3	68.2	489.5	497.8
260	440.0	68.2	533.1	541.4
280	484.1	68.0	577.0	585.4
298.15	524.0	67.8	616.8	625.1
300	528.1	67.8	620.8	629.1
320	571.6	67.6	664.1	672.4
340	614.3	67.2	706.4	714.8
360	656.0	66.8	747.7	756.0
380	696.4	66.3	787.7	796.0

T / K	$C_{v,vib}(T)$ / J K^{-1} mol^{-1} [a]	$\sum C_{v,ir}(T)$ / J K^{-1} mol^{-1} [b]	$C_{v,m}(T)$ / J K^{-1} mol^{-1}	$C_{p,m}(T)$ / J K^{-1} mol^{-1}
400	735.5	65.8	826.3	834.6
420	773.2	65.3	863.4	871.7
440	809.3	64.7	899.0	907.3
460	844.0	64.0	933.0	941.3
480	877.2	63.4	965.5	973.8
500	908.9	62.7	996.5	1004.8
520	939.1	62.0	1026.1	1034.4
540	968.1	61.3	1054.3	1062.6
560	995.7	60.6	1081.2	1089.5
580	1022.1	59.8	1106.9	1115.2
600	1047.3	59.1	1131.3	1139.6
		m-MTDAB		
200	–	–	454.7	463.0
220	–	–	499.2	507.6
240	–	–	545.1	553.4
260	–	–	591.6	599.9
280	–	–	638.4	646.7
298.15	–	–	681.0	689.3
300	–	–	685.2	693.5
320	–	–	731.4	739.7
340	–	–	776.8	785.1
360	–	–	821.2	829.5
380	–	–	864.4	872.7
400	–	–	906.0	914.3
420	–	–	946.2	954.5
440	–	–	984.9	993.2
460	–	–	1022.1	1030.4
480	–	–	1057.4	1065.7
500	–	–	1091.3	1099.6
520	–	–	1123.9	1132.2
540	–	–	1154.9	1163.3
560	–	–	1184.7	1193.0
580	–	–	1213.1	1221.4
600	–	–	1240.2	1248.5

[a] Vibrational contribution to the heat capacity. Calculated based on the set of frequencies listed in Table S15 (column 1) using the equation S6;
[b] Sum of the internal rotation contributions for all rotating tops of the molecule.

7.1 The procedure of ideal gas phase heat capacities calculation

According to the rigid rotor – harmonic oscillator model, the vibrational contribution to the molar heat capacity in the ideal gas state can be calculated by Eq. S6 with the set of frequencies from Table S15:

$$C_{\text{v,vib}} = R \cdot \sum_i \frac{(\frac{\Theta_i}{T})^2 \cdot \exp(\frac{\Theta_i}{T})}{(\exp(\frac{\Theta_i}{T}) - 1)^2}, \qquad \text{(S6)}$$

where Θ_i is the *i*-th fundamental vibrational frequency of the molecule.

The contribution of hindered rotation from each rotating top is calculated by Eq. S7 [19] using the energy levels listed in Table S16:

$$C_{\mathrm{v,ir}} = \frac{\left(\sum_j \exp(-\frac{\varepsilon_j}{kT}) \cdot \sum_j \frac{\varepsilon_j^2}{kT^2} \exp(-\frac{\varepsilon_j}{kT})\right) - \left(\sum_j \frac{\varepsilon_j}{kT^2} \exp(-\frac{\varepsilon_j}{kT}) \cdot \sum_j \varepsilon_j \exp(-\frac{\varepsilon_j}{kT})\right)}{\left(\sum_j \exp(-\frac{\varepsilon_j}{kT})\right)^2}, \quad \text{(S7)}$$

where ε_j is the frequency of the j-th energy level.

The molar isobaric heat capacity in the ideal gas state can be found by summing the contributions of vibration ($C_{\mathrm{v,vib}}$), internal rotation of all rotating tops ($\sum C_{\mathrm{v,ir}}$), translation (3/2 R), overall rotation (3/2 R), and the difference between isobaric and isochoric heat capacity (R):

$$C_{\mathrm{p,m}} = C_{\mathrm{v,vib}} + \sum C_{\mathrm{v,ir}} + C_{\mathrm{v,trn}} + C_{\mathrm{v,rot}} + (C_{\mathrm{p,m}} - C_{\mathrm{v,m}}) = C_{\mathrm{v,vib}} + \sum C_{\mathrm{v,ir}} + 4R \quad \text{(S8)}$$

8 Heat capacity values

Table S19

Ideal gas heat capacities at 298.15 K for organic semiconductors and the corresponding group contributions calculated from these compounds.

**Compound**	**$C_{\mathrm{p,m}}$(g, 298.15 K) / J K^{-1} mol^{-1}**
mCP	415.5
CBP	497.6
TCB	583.2
DDP	443.0
TPB	527.7
TDAB	625.1
p-TTP	485.8
TPD	569.7
m-MTDAB	689.3
**Group**	**Contribution / J K^{-1} mol^{-1}**
benzene	82.4
methyl	21.3
carbazolyl	166.7
diphenylamino	180.6

Table S20

Group contributions to liquid and crystal heat capacities at 298.15 K.

**Group**	**Phase**	**Contribution / J K^{-1} mol^{-1}**
benzene	liquid	136.3
methyl	liquid	8.2
carbazolyl	liquid	234.1
diphenylamino	liquid	264.2
benzene	crystal	93.3
methyl	crystal	20.5
carbazolyl	crystal	177
diphenylamino	crystal	184

9 Organic semiconductors chemical structures

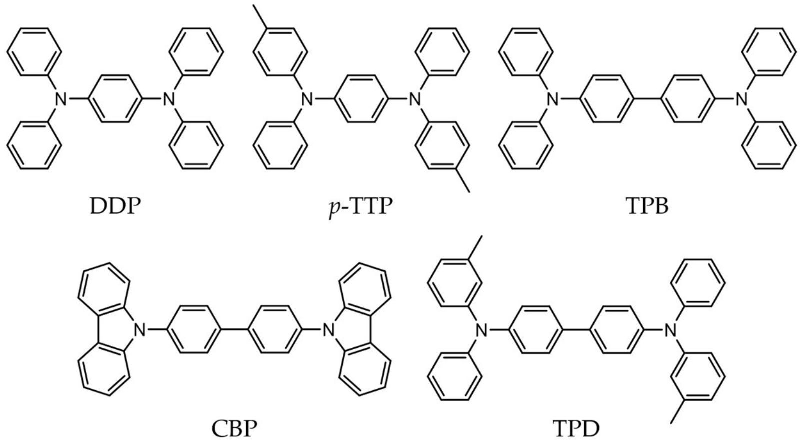

Figure S7. Chemical structures of previously studied DDP (*N,N,N′,N′*-tetraphenyl-*p*-phenylenediamine), *p*-TTP (*N,N′*-diphenyl-*N,N′*-di-*p*-tolylbenzene), TPB (*N,N,N′,N′*-tetraphenylbenzidine), CBP (4,4′-bis(*N*-carbazolyl)-1,1′-biphenyl), and TPD (4,4′-bis(*m*-tolylphenylamino)biphenyl) [26, 27].

References

1. Goursot, P.; Girdhar, H. L.; Westrum Jr, E. F., Thermodynamics of polynuclear aromatic molecules. III. Heat capacities and enthalpies of fusion of anthracene. *The Journal of Physical Chemistry* **1970,** 74, (12), 2538-2541.
2. Grønvold, F., Heat capacity of indium from 300 to 1000 K: Enthalpy of fusion. *Journal of Thermal Analysis and Calorimetry* **1978,** 13, (3), 419-428.
3. Yagofarov, M. I.; Sokolov, A. A.; Gerasimov, A. V.; Solomonov, B. N.; Stepurko, E. N.; Yurkshtovich, Y. N., Thermodynamic Properties of Thioxanthone between 80 and 540 K. *Journal of Chemical & Engineering Data* **2022,** 67, (12), 3583-3588.
4. Höhne, G. W. H.; Hemminger, W.; Flammersheim, H.-J., *Differential scanning calorimetry*. Springer: 2003; Vol. 2.
5. Yagofarov, M. I.; Solomonov, B. N., Calculation of the fusion enthalpy temperature dependence of polyaromatic hydrocarbons from the molecular structure: Old and new approaches. *The Journal of Chemical Thermodynamics* **2021,** 152, 106278.
6. Mukhametzyanov, T. A.; Notfullin, A. A.; Fatkhutdinova, A. A.; Schick, C., Organic compounds as temperature calibrants for Fast Scanning Calorimetry. *Thermochimica Acta* **2024**, 179868.
7. Della Gatta, G.; Richardson, M. J.; Sarge, S. M.; Stølen, S., Standards, calibration, and guidelines in microcalorimetry. Part 2. Calibration standards for differential scanning calorimetry*(IUPAC Technical Report). *Pure and applied chemistry* **2006,** 78, (7), 1455-1476.
8. Yagofarov, M. I.; Sokolov, A. A.; Balakhontsev, I. S.; Nizamov, I. I.; Solomonov, B. N., Thermochemistry of fusion, solution and hydrogen bonding in benzamide, N-methylbenzamide, and acetanilide. *Thermochimica Acta* **2023,** 728, 179579.
9. Donnelly, J.; Drewes, L.; Johnson, R.; Munslow, W.; Knapp, K.; Sovocool, G., Purity and heat of fusion data for environmental standards as determined by differential scanning calorimetry. *Thermochimica acta* **1990,** 167, (2), 155-187.
10. Chang, S.; Bestul, A., Heat Capacity and Thermodynamic Properties of o-Terphenyl Crystal, Glass, and Liquid. *The Journal of Chemical Physics* **1972,** 56, (1), 503-516.
11. Štejfa, V.; Pokorný, V.; Mathers, A.; Růžička, K.; Fulem, M., Heat capacities of selected active pharmaceutical ingredients. *The Journal of Chemical Thermodynamics* **2021,** 163, 106585.
12. Buzyurov, A. V.; Nagrimanov, R. N.; Zaitsau, D. H.; Mukhametzyanov, T. A.; Solomonov, B. N.; Abdelaziz, A.; Schick, C., Application of the Flash DSC 1 and 2+ for vapor pressure determination above solids and liquids. *Thermochimica Acta* **2021,** 706, 179067.

13. Růůžička, K.; Mokbel, I.; Majer, V.; Růůžička, V.; Jose, J.; Zábranský, M., Description of vapour–liquid and vapour–solid equilibria for a group of polycondensed compounds of petroleum interest. *Fluid phase equilibria* **1998,** 148, (1-2), 107-137.
14. Zaitsau, D. H.; Pimerzin, A. A.; Verevkin, S. P., Fatty acids methyl esters: Complementary measurements and comprehensive analysis of vaporization thermodynamics. *The Journal of Chemical Thermodynamics* **2019,** 132, 322-340.
15. van Bommel, M. J.; Oonk, H. A.; van Miltenburg, J. C., Heat capacity measurements of 13 methyl esters of n-carboxylic acids from methyl octanoate to methyl eicosanoate between 5 K and 350 K. *Journal of Chemical & Engineering Data* **2004,** 49, (4), 1036-1042.
16. Finke, H.; Messerly, J.; Lee, S.; Osborn, A.; Douslin, D., Comprehensive thermodynamic studies of seven aromatic hydrocarbons. *The Journal of Chemical Thermodynamics* **1977,** 9, (10), 937-956.
17. Hallén, D.; Nilsson, S.-O.; Rothschild, W.; Wadsö, I., Enthalpies and heat capacities for n-alkan-1-ols in H2O and D2O. *The Journal of Chemical Thermodynamics* **1986,** 18, (5), 429-442.
18. Group, I. W.; Sabbah, R.; Xu-wu, A.; USA, J. C.; Leitão, M. P.; Roux, M.; Torres, L., Reference materials for calorimetry and differential thermal analysis. *Thermochimica Acta* **1999,** 331, (2), 93-204.
19. Pfaendtner, J.; Yu, X.; Broadbelt, L. J., The 1-D hindered rotor approximation. *Theoretical Chemistry Accounts* **2007,** 118, 881-898.
20. Kilpatrick, J. E.; Pitzer, K. S., Energy levels and thermodynamic functions for molecules with internal rotation. III. Compound rotation. *The Journal of Chemical Physics* **1949,** 17, (11), 1064-1075.
21. Balint-Kurti, G. G.; Dixon, R. N.; Marston, C. C., Grid methods for solving the Schrödinger equation and time dependent quantum dynamics of molecular photofragmentation and reactive scattering processes. *International Reviews in Physical Chemistry* **1992,** 11, (2), 317-344.
22. Balint-Kurti, G. G.; Ward, C. L.; Marston, C. C., Two computer programs for solving the Schrödinger equation for bound-state eigenvalues and eigenfunctions using the Fourier grid Hamiltonian method. *Computer physics communications* **1991,** 67, (2), 285-292.
23. Marston, C. C.; Balint-Kurti, G. G., The Fourier grid Hamiltonian method for bound state eigenvalues and eigenfunctions. *The Journal of chemical physics* **1989,** 91, (6), 3571-3576.
24. nist, Proga ot nista. *1* **1,** 1, (1), 1.
25. Ayala, P. Y.; Schlegel, H. B., Identification and treatment of internal rotation in normal mode vibrational analysis. *The Journal of chemical physics* **1998,** 108, (6), 2314-2325.
26. Bolmatenkov, D. N.; Notfullin, A. A.; Sokolov, A. A.; Balakhontsev, I. S.; Yagofarov, M. I.; Mukhametzyanov, T. A.; Solomonov, B. N., Phase transition thermodynamics of organic semiconductors: N, N, N′, N′-tetraphenylbenzidine and 4, 4′-bis (N-carbazolyl)-1, 1′-biphenyl. *Journal of Molecular Liquids* **2024,** 403, 124810.
27. Notfullin, A. A.; Bolmatenkov, D. N.; Sokolov, A. A.; Balakhontsev, I. S.; Kachmarzhik, A. D.; Solomonov, B. N.; Yagofarov, M. I., Phase transition thermodynamics of organic semiconductors N, N, N′, N′-tetraphenyl-p-phenylenediamine, N, N′-diphenyl-N, N′-di-p-tolylbenzene-1, 4-diamine, and 4, 4′-bis (m-tolylphenylamino) biphenyl. *The Journal of Chemical Thermodynamics* **2025,** 206, 107470.

↑
